# Supplementary material for: Natural plant growth and development achieved in the IPK PhenoSphere by dynamic environment simulation
Source: Nat Commun. 2023 Sep 18;14:5783. doi: 10.1038/s41467-023-41332-4 (PMC10507097; doi:10.1038/s41467-023-41332-4)
Supplement: Supplementary file 1 — Supplementary Information [file 41467_2023_41332_MOESM1_ESM.pdf]

Supplementary Information

Natural plant growth and development achieved in the IPK PhenoSphere by dynamic environment simulation

Marc C. Heuermann[1], Dominic Knoch[1], Astrid Junker[1,2], and Thomas Altmann[1]

[1] Department of Molecular Genetics, Leibniz Institute of Plant Genetics and Crop Plant Research (IPK), Corrensstrasse 3, 06466 Seeland OT Gatersleben, Germany

[2] present address: Syngenta Seeds GmbH, Zum Knipkenbach 20, 32107 Bad Salzuflen, Germany

Supplementary Figure 1 - page 2
Supplementary Figure 2 - page 3
Supplementary Figure 3 - page 4
Supplementary Figure 4 - page 5
Supplementary Figure 5 - page 6
Supplementary Figure 6 - page 7
Supplementary Figure 7 - page 8
Supplementary Figure 8 - page 9
Supplementary Figure 9 - page 10
Supplementary Figure 10 - page 11
Supplementary Figure 11 - page 12
Supplementary Figure 12 - page 13
Supplementary Figure 13 - page 14
Supplementary Figure 14 - page 15
Supplementary Figure 15 - page 16
Supplementary Figure 16 - page 17
Supplementary Figure 17 - page 18
Supplementary Figure 18 - page 19, 20, 21
Supplementary Figure 19 - page 22
Supplementary Figure 20 - page 23
Supplementary Figure 21 - page 24
Supplementary Figure 22 - page 25, 26

Supplementary Table 1 - page 27
Supplementary Table 2 - page 28
Supplementary Table 3 - page 29, 30
Supplementary Table 4 - page 31
Supplementary Table 5 - page 32
Supplementary Table 6 - page 33

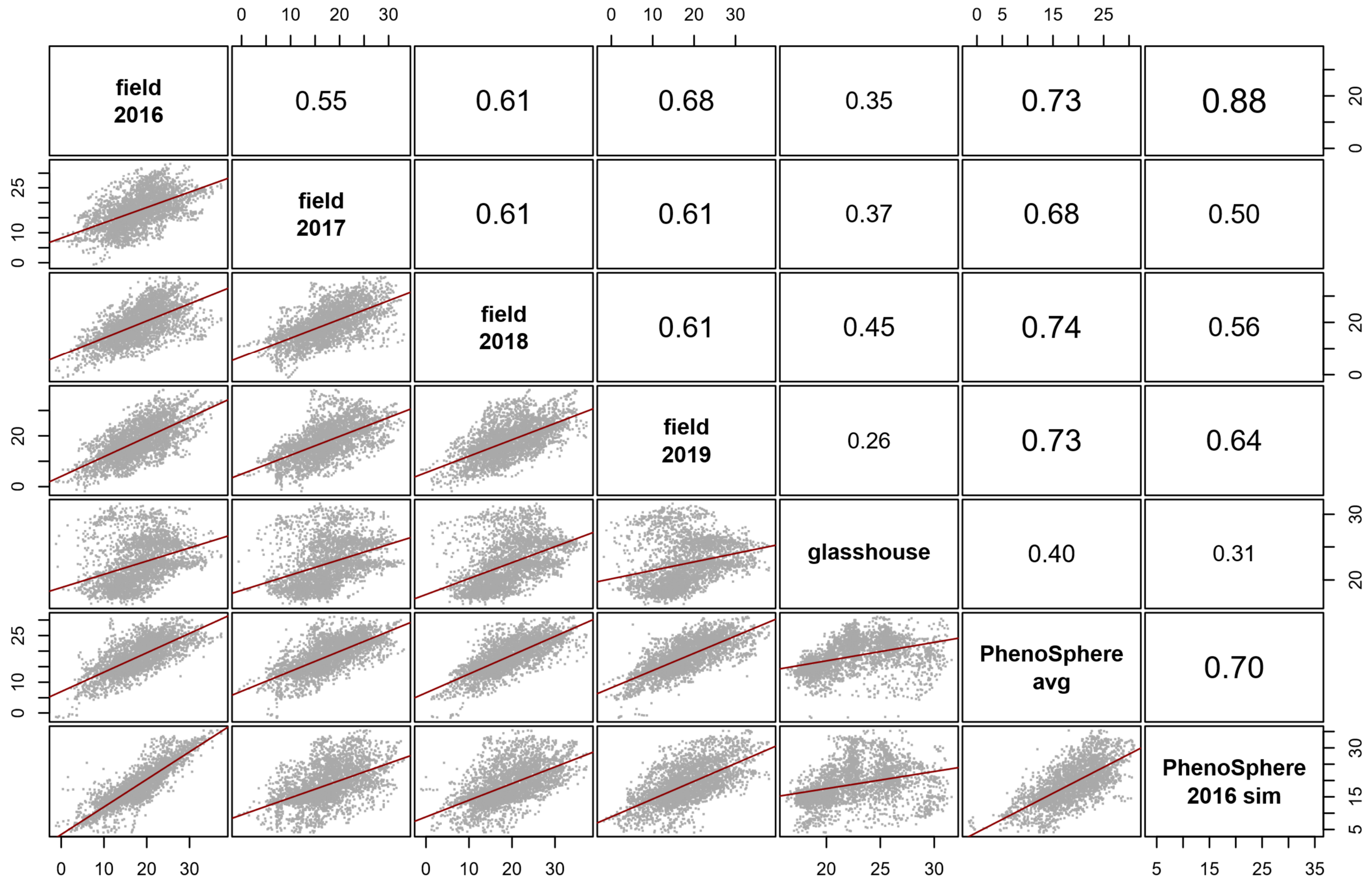

Supplementary Figure 1: **Correlogram of the temperature profiles [°C] measured in the seven environments (2nd May – 13th September).** The lower panel shows the data distribution with a regression line, and the upper panel provides the corresponding Pearson correlation coefficients 'r'. All correlations were significant (two-sided) and had a p-value < 2.2e-16.

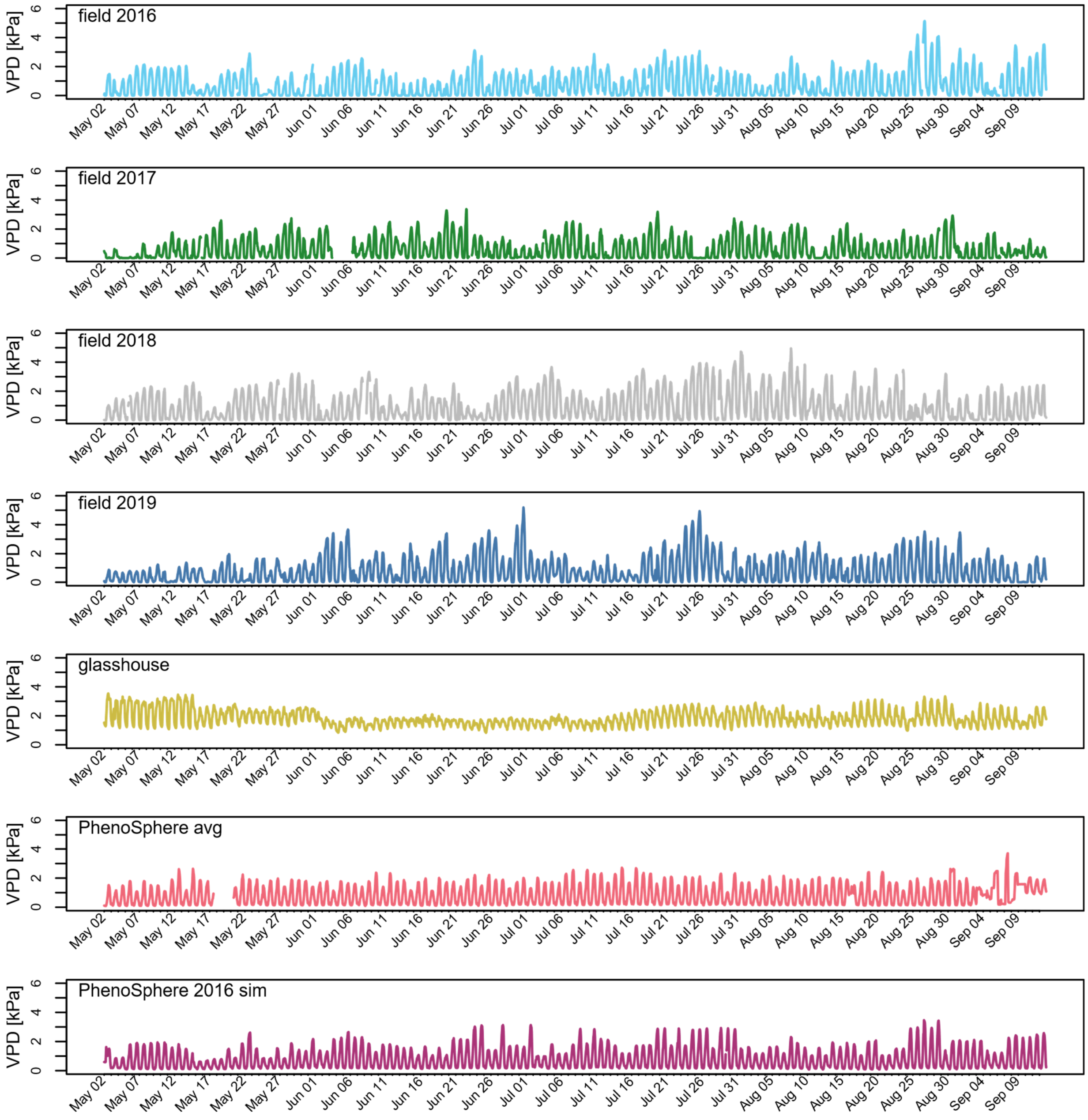

Supplementary Figure 2: **Vapor pressure deficit (VPD) profiles [kPa] of all seven environments (2nd May – 13th September).** VPD in the field 2016 – 2019, the glasshouse, the 'PhenoSphere avg', and the 'PhenoSphere 2016 sim' experiments. VPD values were plotted in an hourly resolution except for minor gaps due to sensor failures. Dates for the field experiments are true calendar dates and the dates in the controlled environment have been relatively mapped to simulate their templates. Colors used: field 2016 as Sky Blue (#66CCEE), field 2017 as Forest Green (#228833), field 2018 as Silver (#BBBBBB), field 2019 as San Marino (#4477AA), glasshouse as Turmeric (#CCBB44), PhenoSphere avg as Froly (#EE6677), and PhenoSphere 2016 sim as Royal Heath (#AA3377).

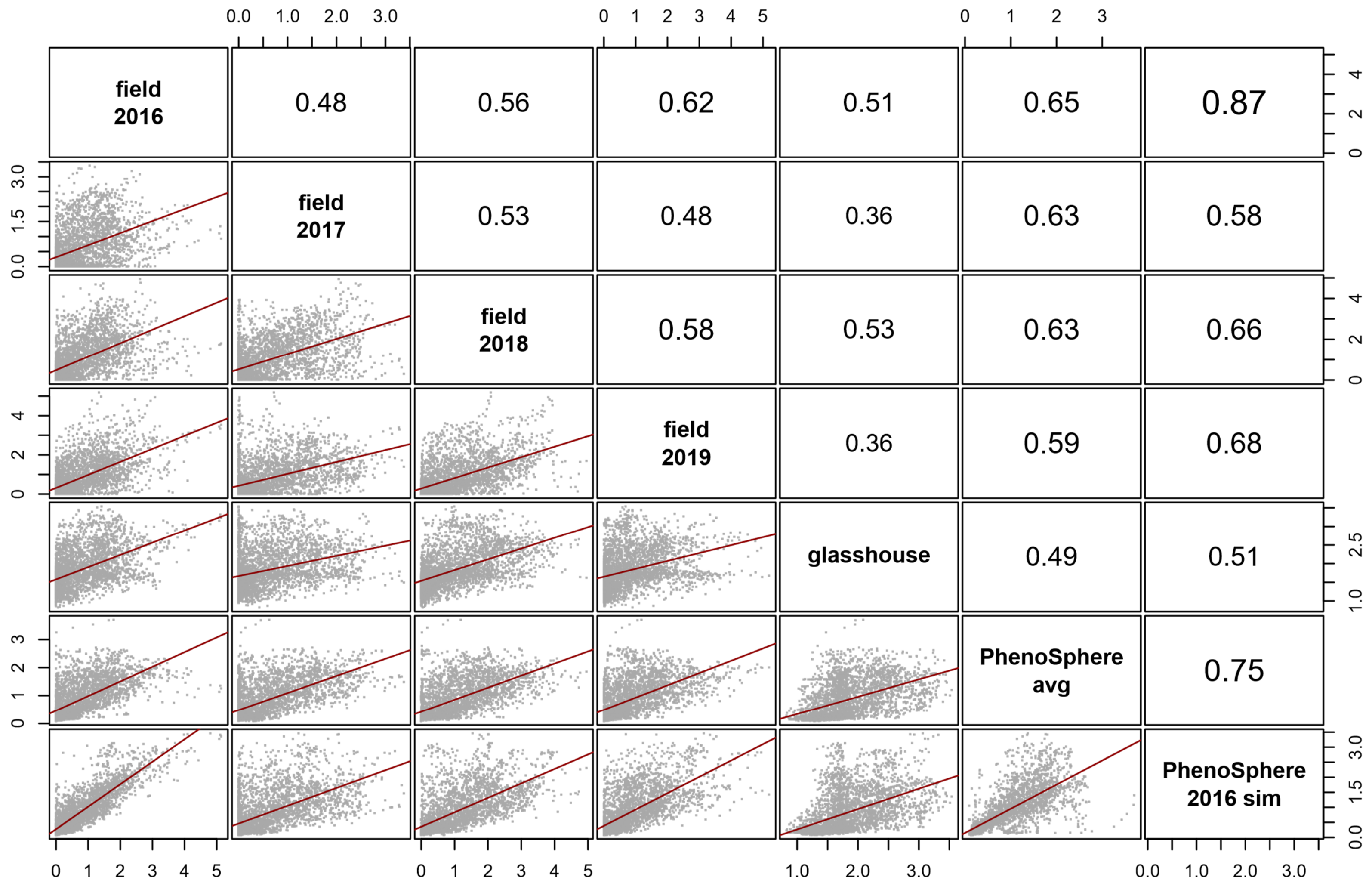

Supplementary Figure 3: **Correlogram of the vapor pressure deficit (VPD) profiles [kPa] measured in the seven environments (2nd May – 13th September).** The lower panel shows the data distribution with a regression line, and the upper panel provides the corresponding Pearson correlation coefficients ‘r’. All correlations were significant (two-sided) and had a p-value < 2.2e-16.

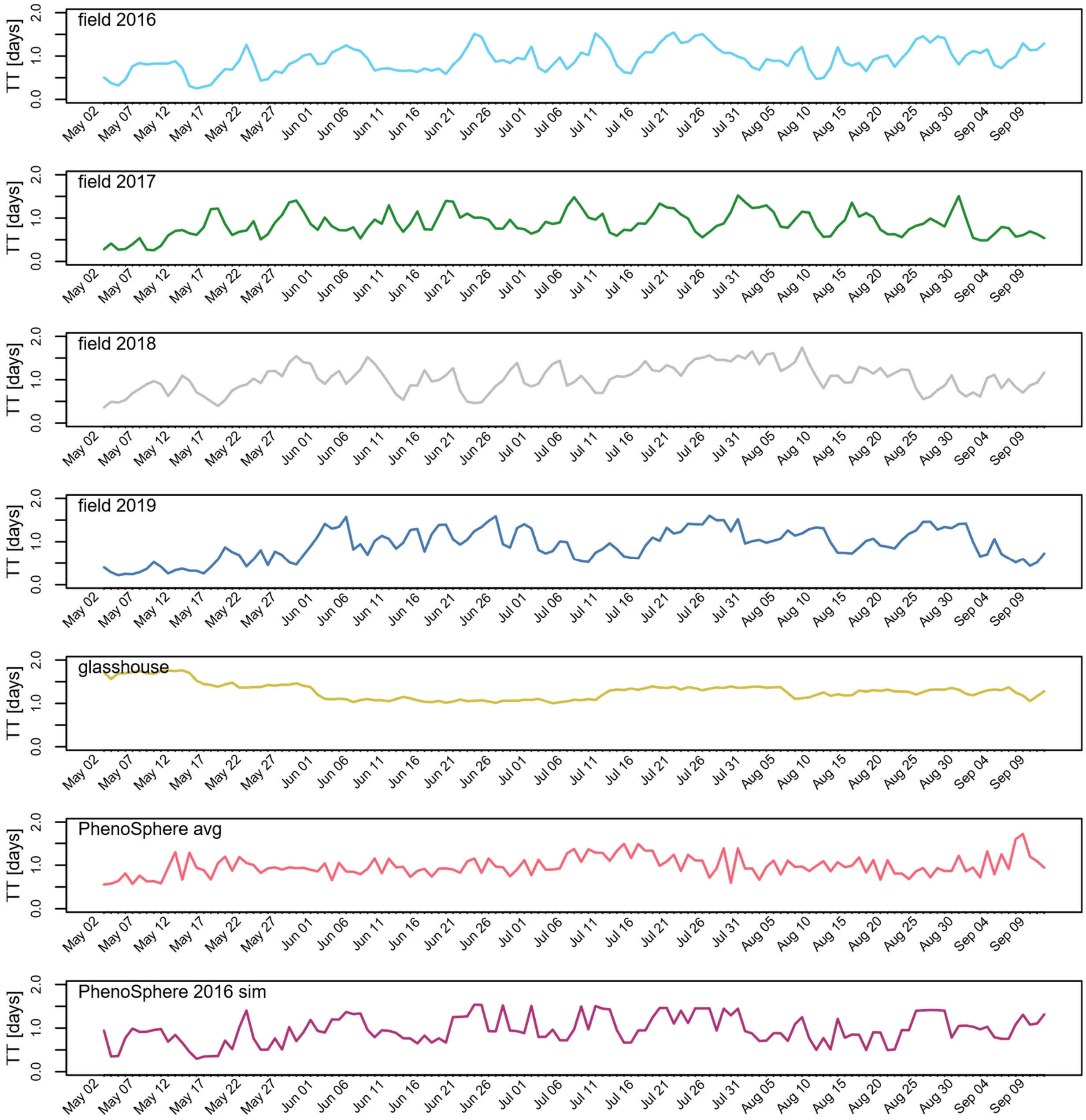

Supplementary Figure 4: Daily cumulative thermal time (TT) contribution of real calendar dates in each environment (2nd May – 13th September). TT in the field 2016 – 2019, the glasshouse, the ‘PhenoSphere avg’, and the ‘PhenoSphere 2016 sim’ experiments. Thermal time days were plotted in a daily resolution. Dates for the field experiments are true calendar dates and the dates in the controlled environment have been relatively mapped to simulate their templates. Colors used: field 2016 as Sky Blue (#66CCEE), field 2017 as Forest Green (#228833), field 2018 as Silver (#BBBBBB), field 2019 as San Marino (#4477AA), glasshouse as Turmeric (#CCBB44), PhenoSphere avg as Froly (#EE6677), and PhenoSphere 2016 sim as Royal Heath (#AA3377).

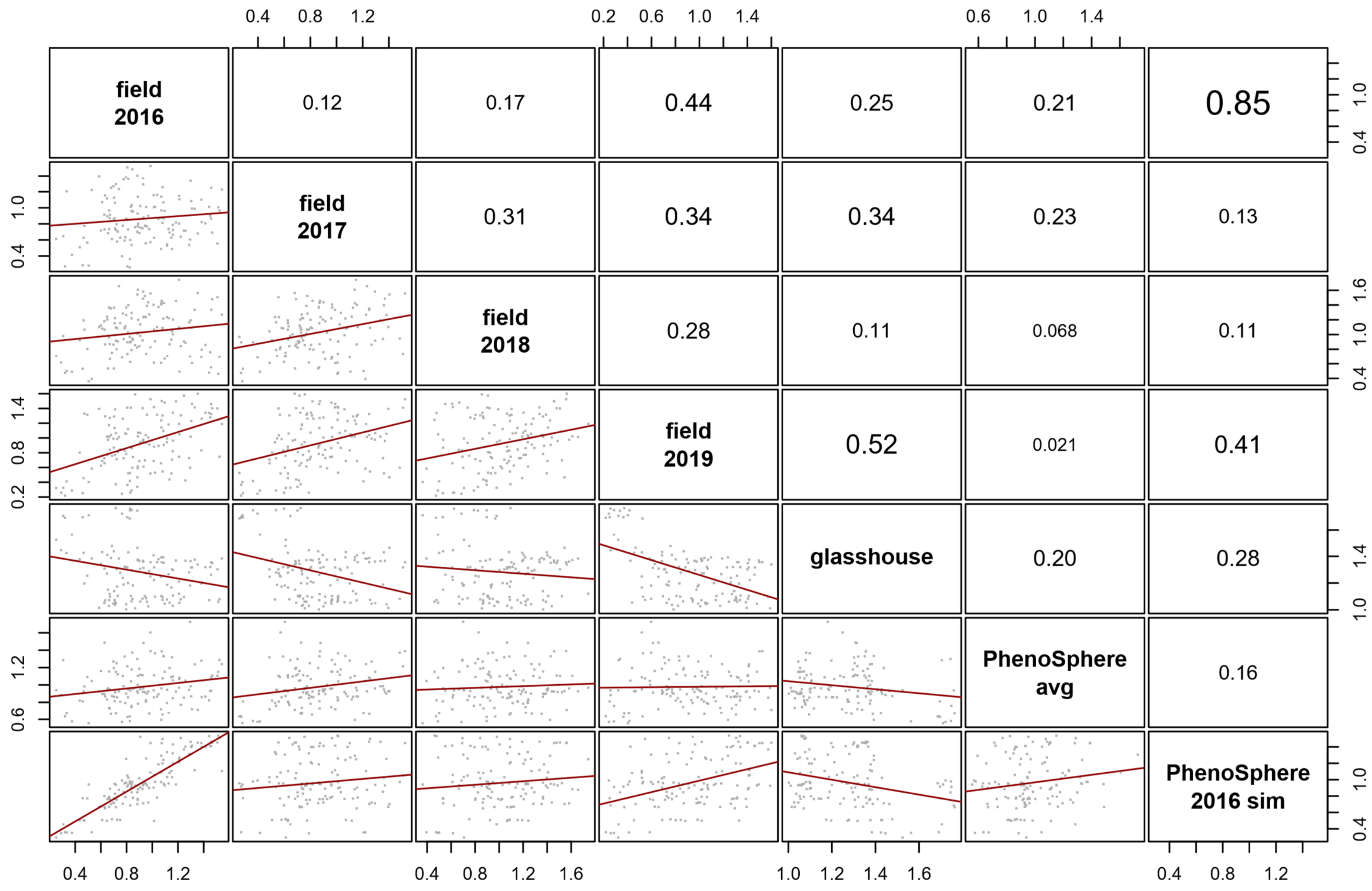

Supplementary Figure 5: **Correlogram of the daily cumulative thermal time contribution measured in the seven environments (2nd May – 13th September).** The lower panel shows the data distribution with a regression line, and the upper panel provides the absolute corresponding Pearson correlation coefficients ‘r’. All correlations (two-sided) with a correlation coefficient > 0.2 were significant p < 0.05. The correlation between field 2016 and PhenoSphere 2016 sim had a p-value < 2.2e-16.

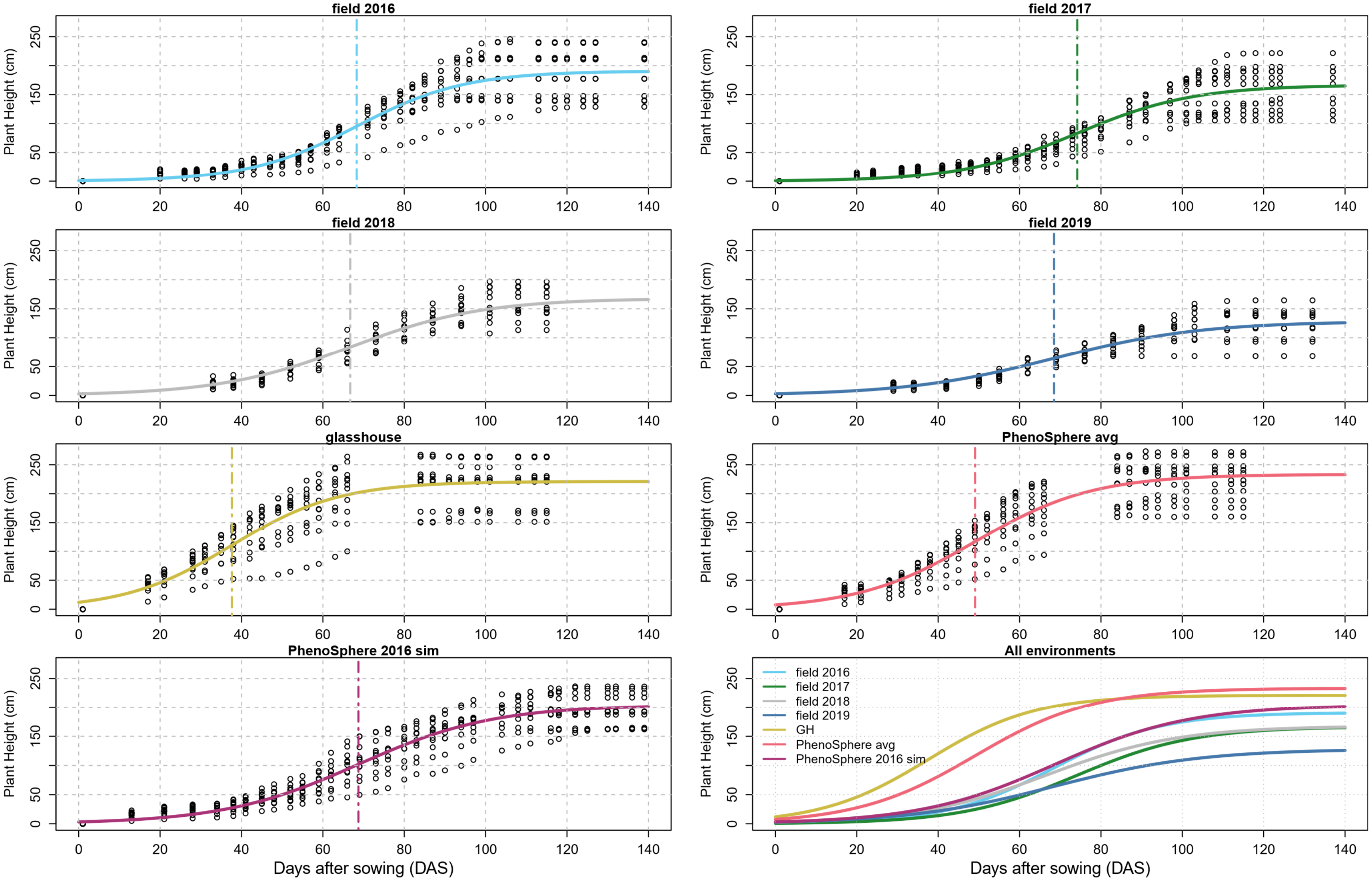

Supplementary Figure 6: **The best fit nonlinear logistic growth model of each environment to the BLUEs of the plant height of all genotypes over time (days after sowing; DAS).** Vertical dashed lines show the 'xmid' parameter, the days after sowing (DAS), when the inflection point of the logistic growth curve, the growth speed, was maximal. Colors used: field 2016 as Sky Blue (#66CCEE), field 2017 as Forest Green (#228833), field 2018 as Silver (#BBBBBB), field 2019 as San Marino (#4477AA), glasshouse as Turmeric (#CCBB44), PhenoSphere avg as Froly (#EE6677), and PhenoSphere 2016 sim as Royal Heath (#AA3377).

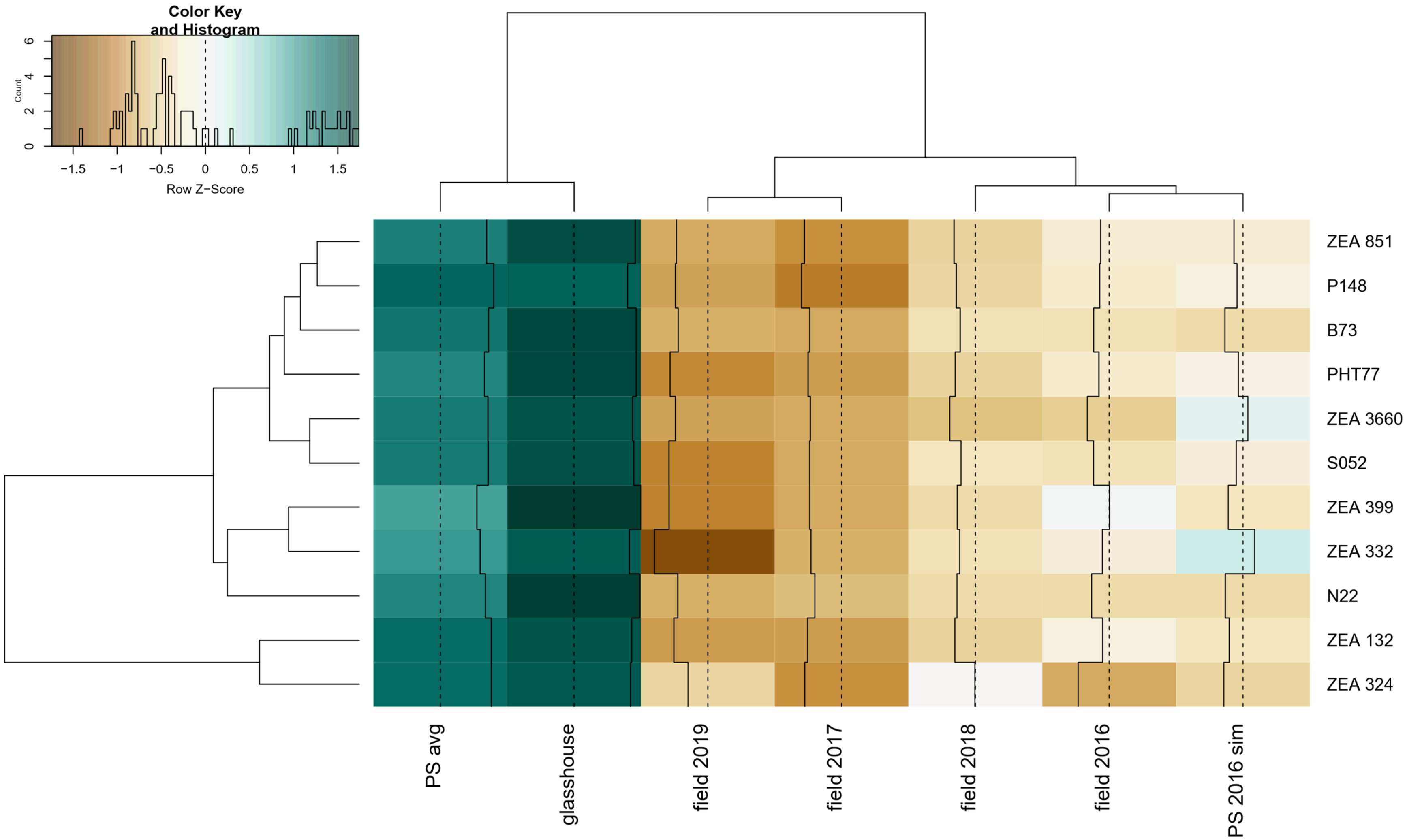

Supplementary Figure 7: Hierarchical clustering of the plant height values from the logistic growth function solved for 68 days after sowing (DAS). Plant height in the field 2016 – 2019, the glasshouse, the PhenoSphere avg (PS avg), and the PhenoSphere 2016 sim (PS 2016 sim). Complete-linkage clustering was performed on the Manhattan distance matrix and scaled for rows. Colors represent the z-score calculated for rows.

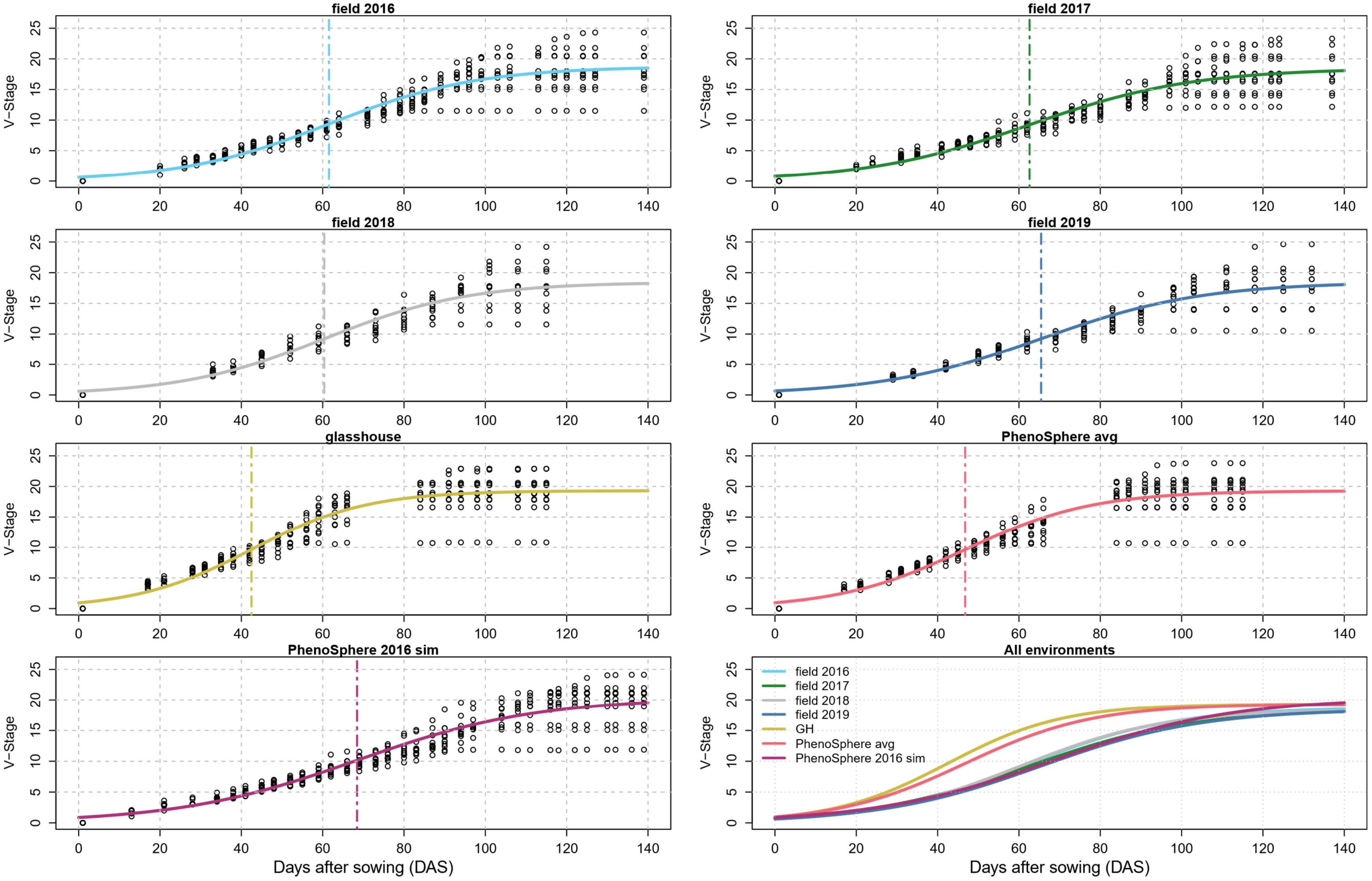

Supplementary Figure 8: **The best fit nonlinear logistic growth model of each environment to the BLUEs of the vegetative stage (V-stage) of all genotypes over time (days after sowing; DAS).** Vertical dashed lines show the 'xmid' parameter, the days after sowing (DAS), when the inflection point of the logistic growth curve, the rate of leaf maturation, was maximal. Colors used: field 2016 as Sky Blue (#66CCEE), field 2017 as Forest Green (#228833), field 2018 as Silver (#BBBBBB), field 2019 as San Marino (#4477AA), glasshouse as Turmeric (#CCBB44), PhenoSphere avg as Froly (#EE6677), and PhenoSphere 2016 sim as Royal Heath (#AA3377).

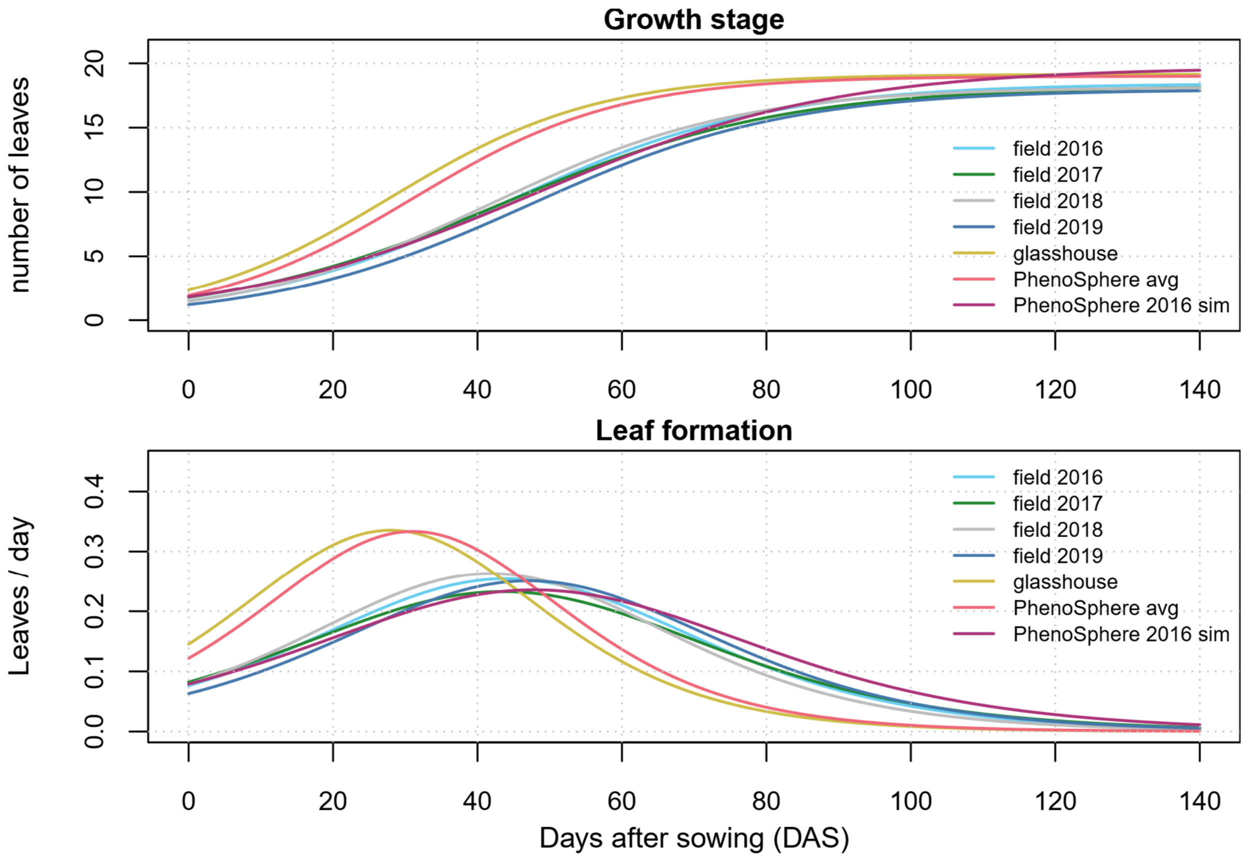

Supplementary Figure 9: **Growth stage plotted as a best fit logistic growth curve (for fit to BLUEs see Supplementary Fig. 10) representing the number of total leaves of the maize population in each environment versus days after sowing (DAS).** The first derivative of the fitted curve representing change in leaf development per DAS for each environment. Colors used: field 2016 as Sky Blue (#66CCEE), field 2017 as Forest Green (#228833), field 2018 as Silver (#BBBBBB), field 2019 as San Marino (#4477AA), glasshouse as Turmeric (#CCBB44), PhenoSphere avg as Froly (#EE6677), and PhenoSphere 2016 sim as Royal Heath (#AA3377).

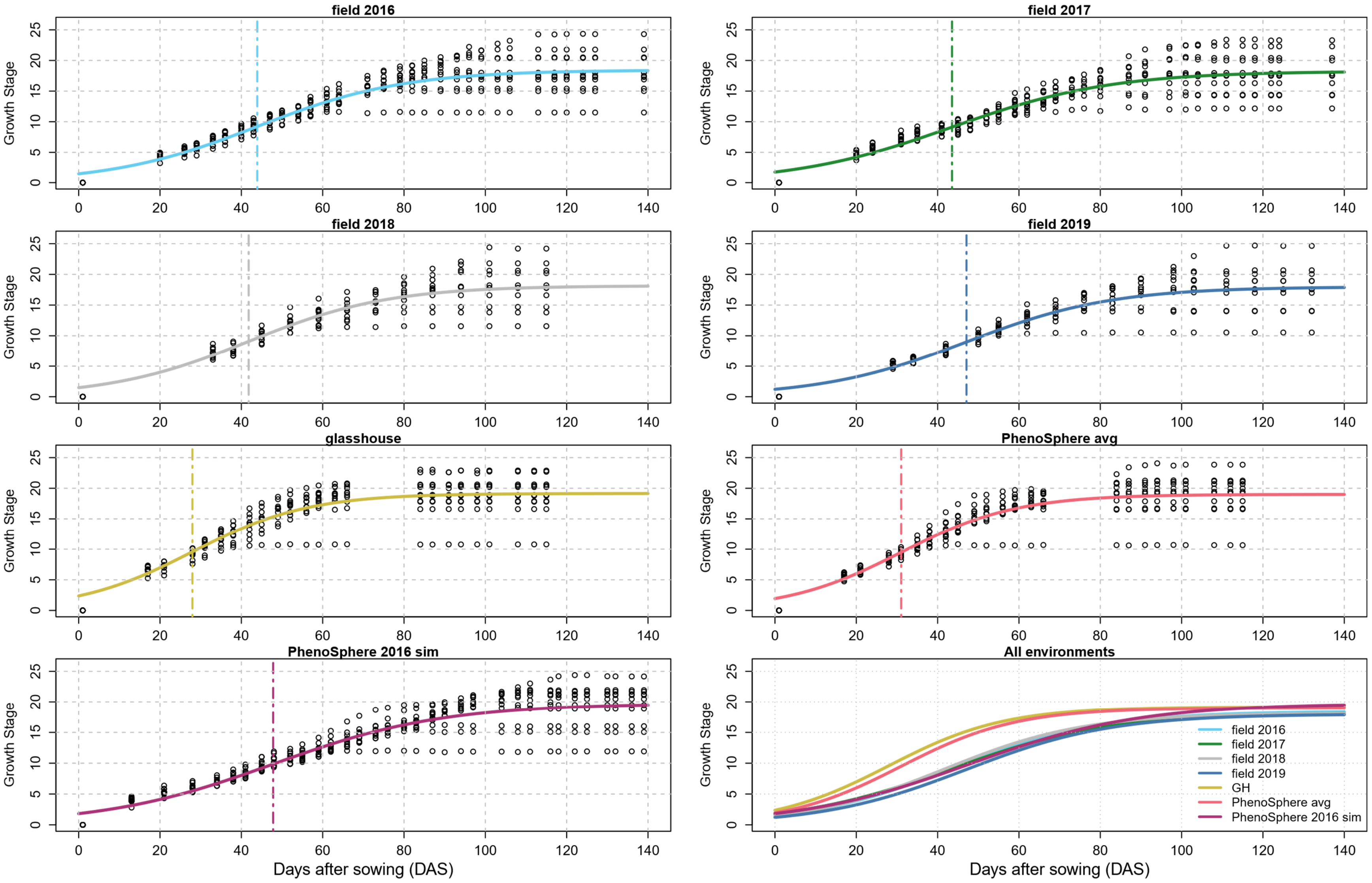

Supplementary Figure 10: The best fit nonlinear logistic growth model of each environment to the BLUEs of the growth stage, counting all leaves, of all genotypes over time (days after sowing; DAS). Vertical dashed lines show the ‘xmid’ parameter, the days after sowing (DAS), when the inflection point of the logistic growth curve was maximal. Colors used: field 2016 as Sky Blue (#66CCEE), field 2017 as Forest Green (#228833), field 2018 as Silver (#BBBBBB), field 2019 as San Marino (#4477AA), glasshouse as Turmeric (#CCBB44), PhenoSphere avg as Froly (#EE6677), and PhenoSphere 2016 sim as Royal Heath (#AA3377).

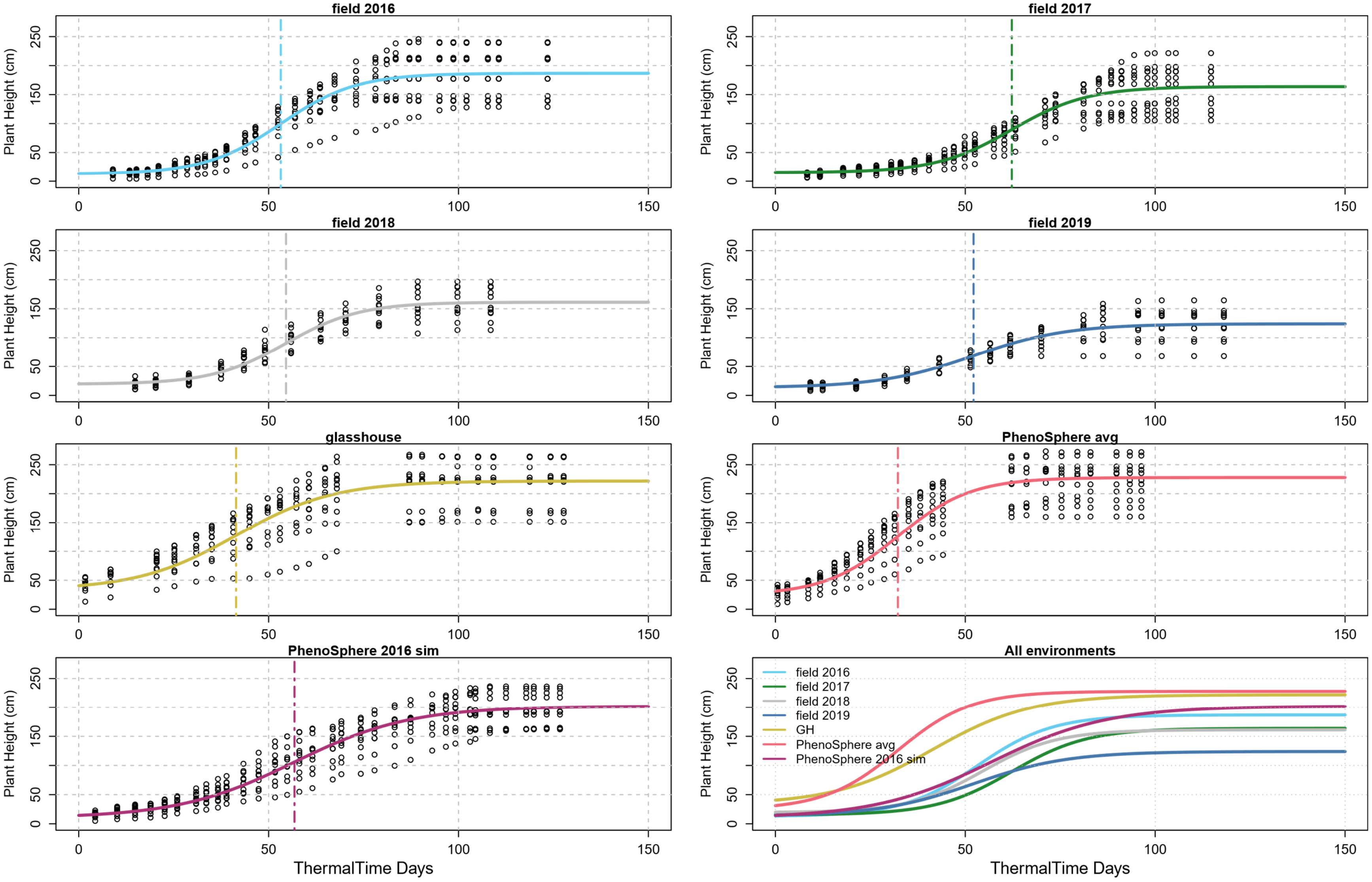

Supplementary Figure 11: **The best fit nonlinear logistic growth model of each environment to the BLUEs of the plant height of all genotypes over thermal time days (temperature converted to thermal time days).** Vertical dashed lines show the 'xmid' parameter, the thermal time days, when the inflection point of the logistic growth curve, the growth speed, was maximal. Colors used: field 2016 as Sky Blue (#66CCEE), field 2017 as Forest Green (#228833), field 2018 as Silver (#BBBBBB), field 2019 as San Marino (#4477AA), glasshouse as Turmeric (#CCBB44), PhenoSphere avg as Froly (#EE6677), and PhenoSphere 2016 sim as Royal Heath (#AA3377).

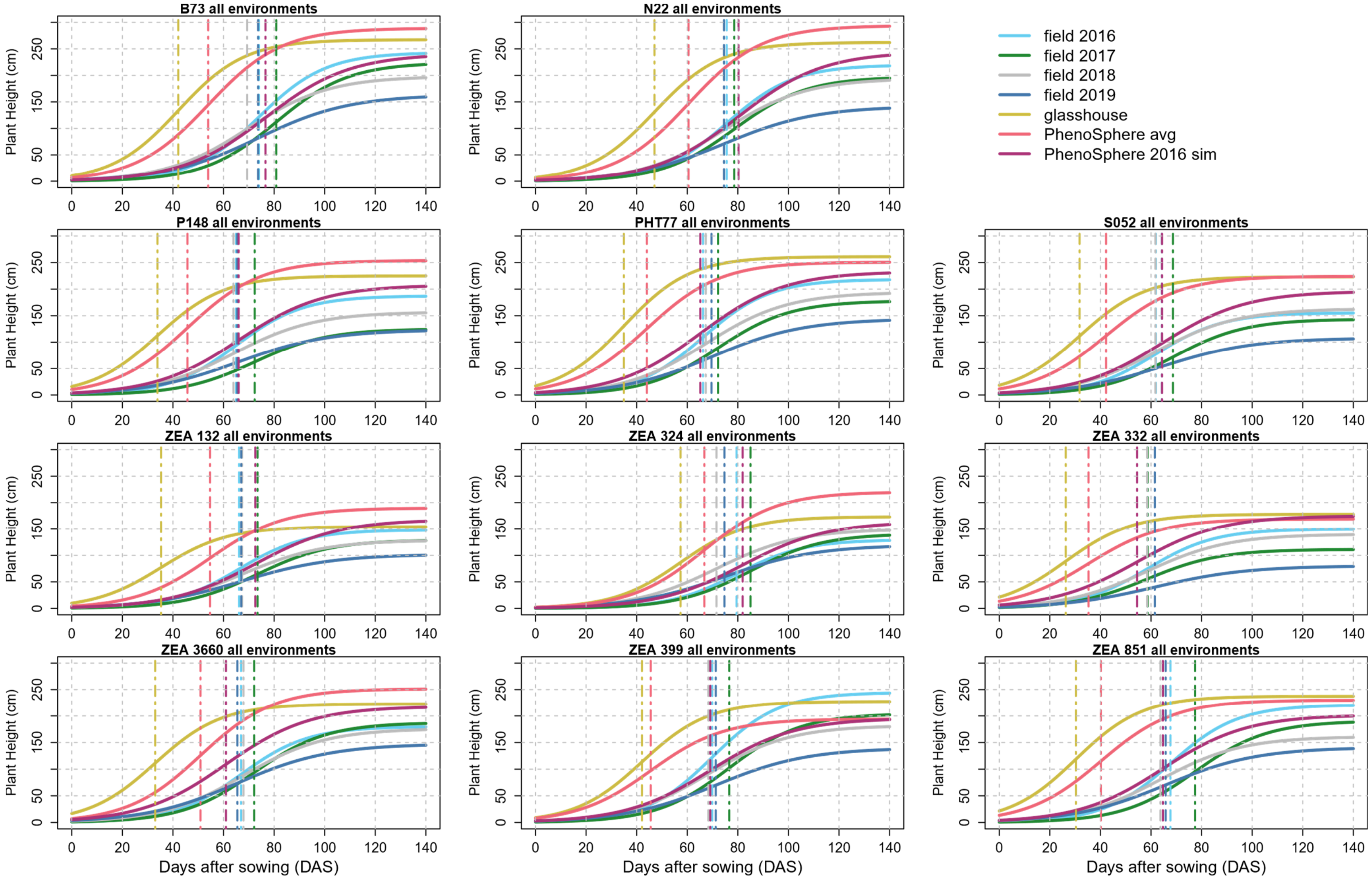

Supplementary Figure 12: **Temporal development of plant height for each genotype individually over all seven environments.** Temporal development was derived from the best fit logistic growth model from the random effects “Asym” and “xmid” and from the common fixed effect “scal”. Vertical dashed lines show the individual “xmid” values for each environment genotype wise. Colors used: field 2016 as Sky Blue (#66CCEE), field 2017 as Forest Green (#228833), field 2018 as Silver (#BBBBBB), field 2019 as San Marino (#4477AA), glasshouse as Turmeric (#CCBB44), PhenoSphere avg as Froly (#EE6677), and PhenoSphere 2016 sim as Royal Heath (#AA3377).

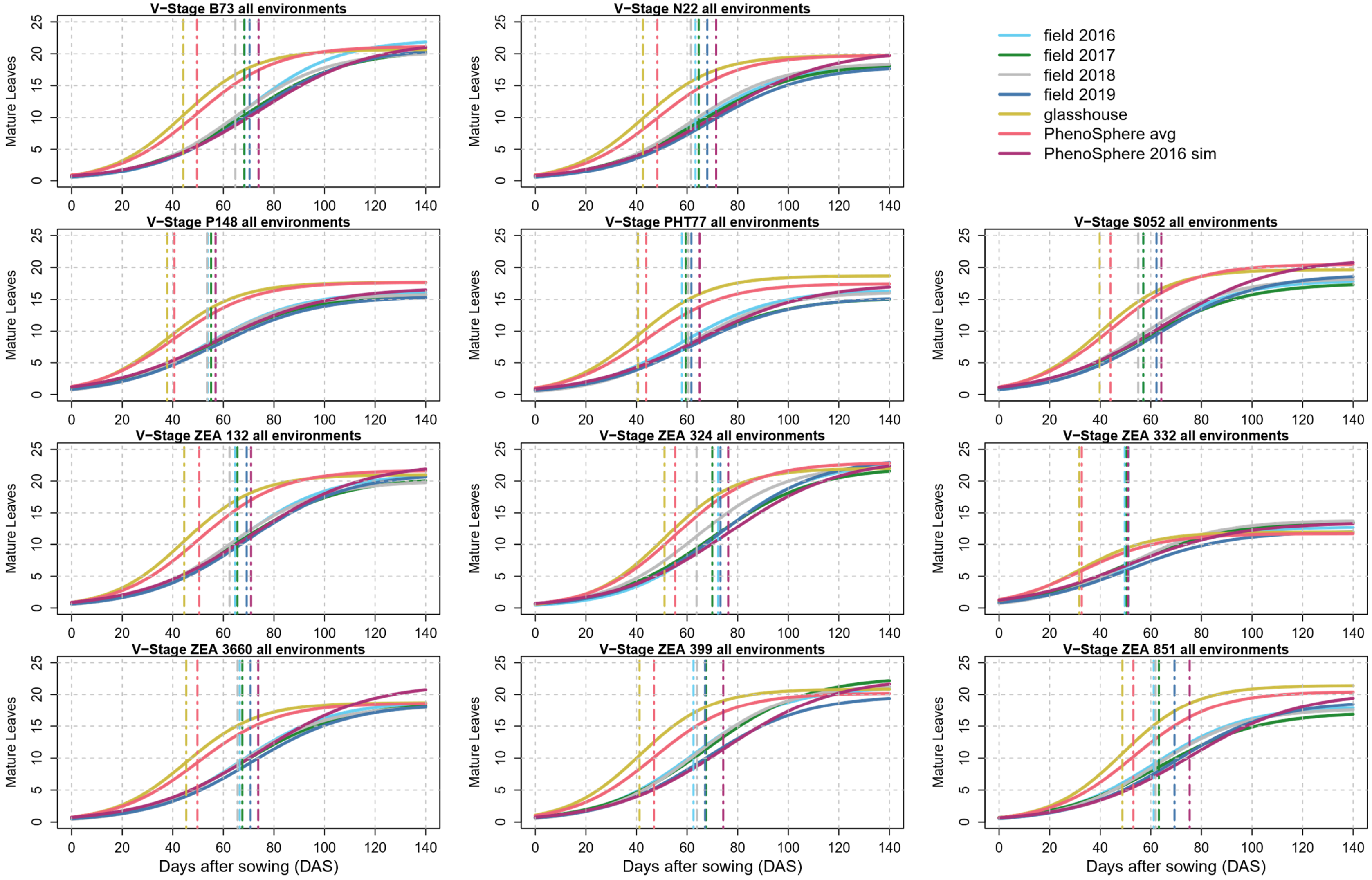

Supplementary Figure 13: **Temporal development of vegetative stage (V-stage) for each genotype individually over all seven environments.** Temporal development was derived from the best fit logistic growth model from the random effects “Asym” and “xmid” and from the common fixed effect “scal”. Vertical dashed lines show the individual “xmid” values for each environment genotype wise. Colors used: field 2016 as Sky Blue (#66CCEE), field 2017 as Forest Green (#228833), field 2018 as Silver (#BBBBBB), field 2019 as San Marino (#4477AA), glasshouse as Turmeric (#CCBB44), PhenoSphere avg as Froly (#EE6677), and PhenoSphere 2016 sim as Royal Heath (#AA3377).

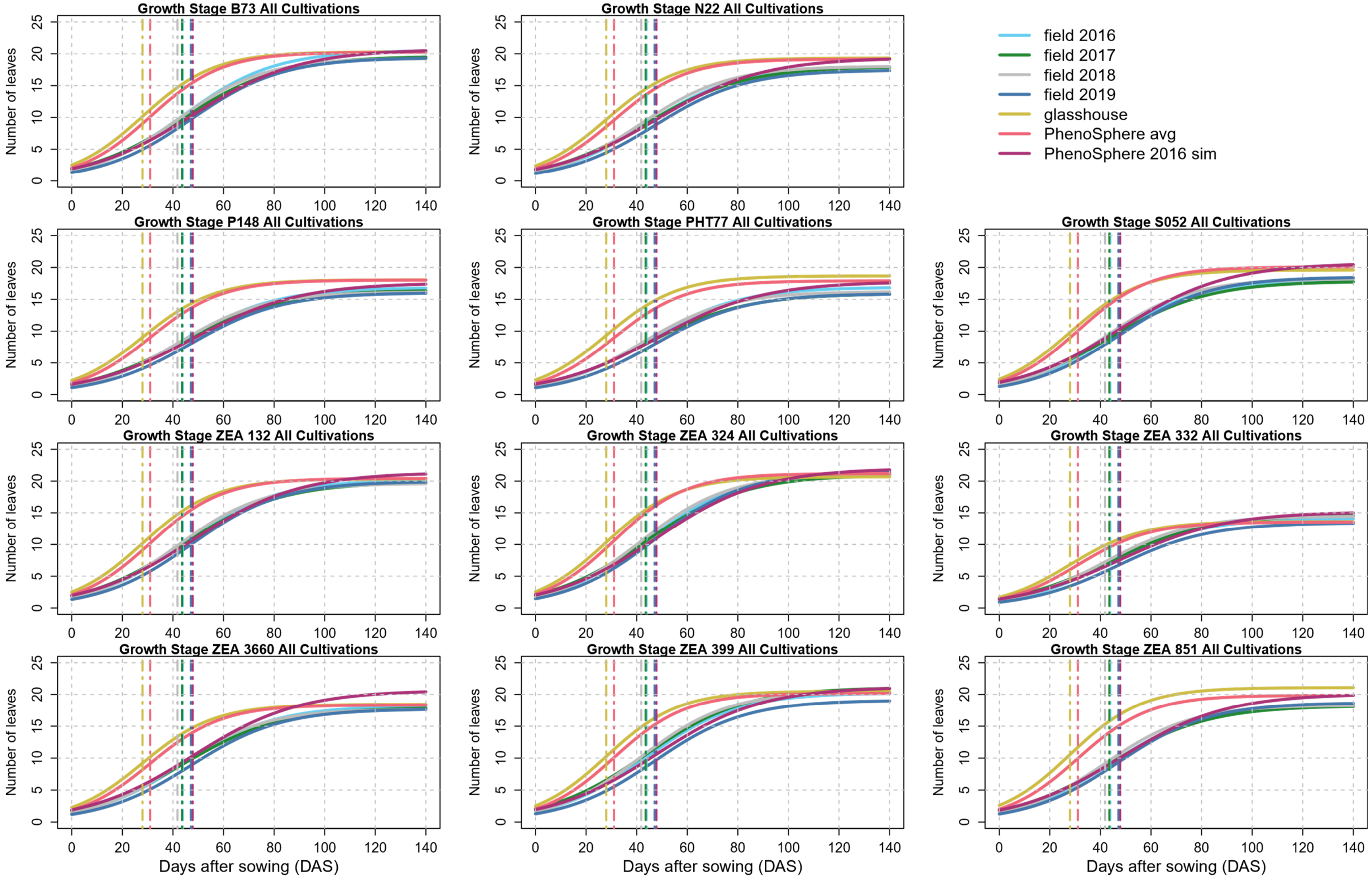

Supplementary Figure 14: **Temporal development of growth stage for each genotype individually over all seven environments.** Temporal development was derived from the best fit logistic growth model from the random effects “Asym” and from the common fixed effect “scal” and “xmid”. Vertical dashed lines show the common fixed-effect “xmid” values for each environment but not solvable for each genotype. Colors used: field 2016 as Sky Blue (#66CCEE), field 2017 as Forest Green (#228833), field 2018 as Silver (#BBBBBB), field 2019 as San Marino (#4477AA), glasshouse as Turmeric (#CCBB44), PhenoSphere avg as Froly (#EE6677), and PhenoSphere 2016 sim as Royal Heath (#AA3377).

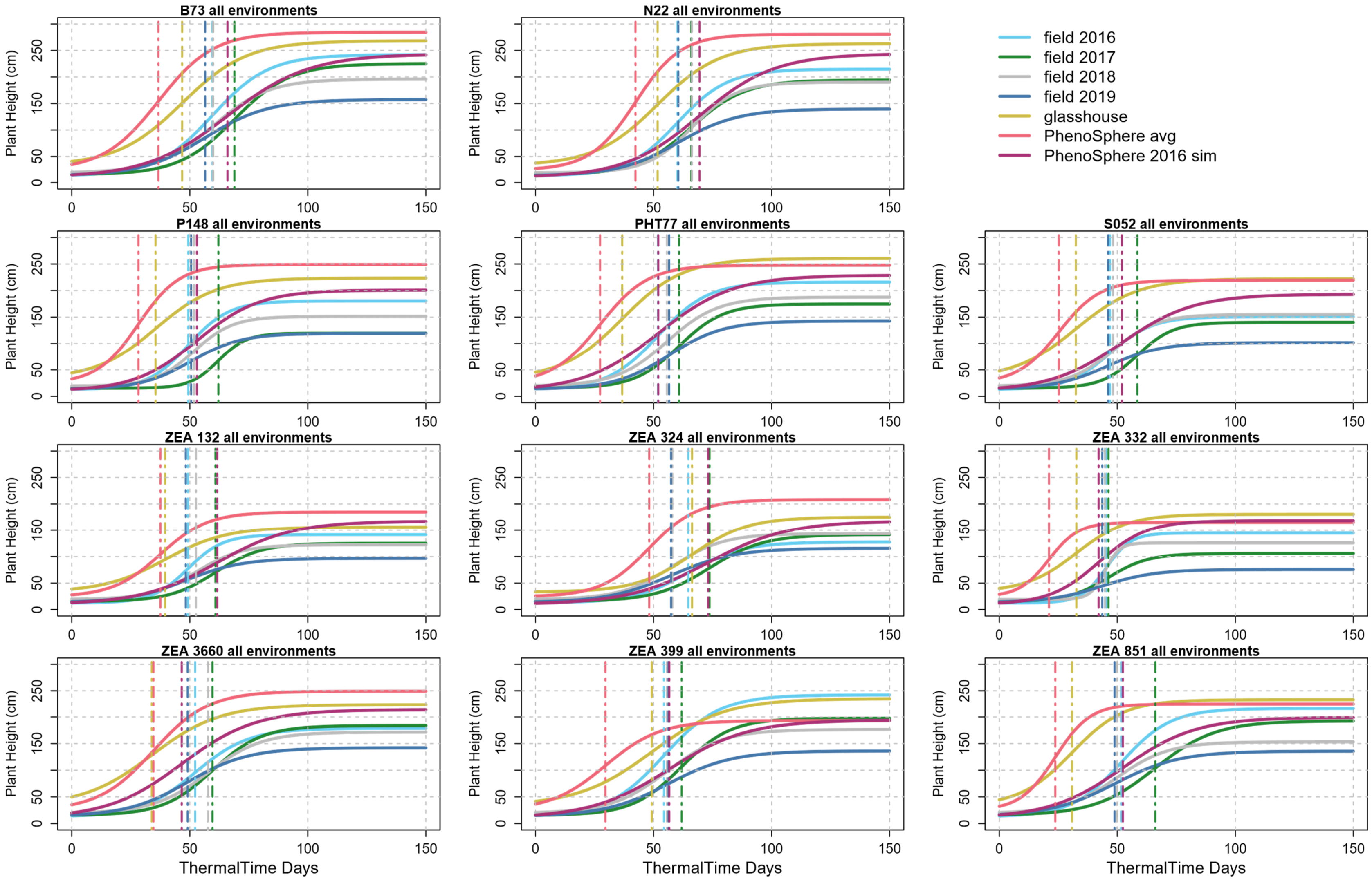

Supplementary Figure 15: **Thermal time (TT) days dependent development of plant height for each genotype individually over all seven environments.** TT development was derived from the best fit four-parameter logistic growth model from the random-effects “A”, “B”, “scal” and “xmid”. Vertical dashed lines show the individual “xmid” values for each environment genotype wise. Colors used: field 2016 as Sky Blue (#66CCEE), field 2017 as Forest Green (#228833), field 2018 as Silver (#BBBBBB), field 2019 as San Marino (#4477AA), glasshouse as Turmeric (#CCBB44), PhenoSphere avg as Froly (#EE6677), and PhenoSphere 2016 sim as Royal Heath (#AA3377).

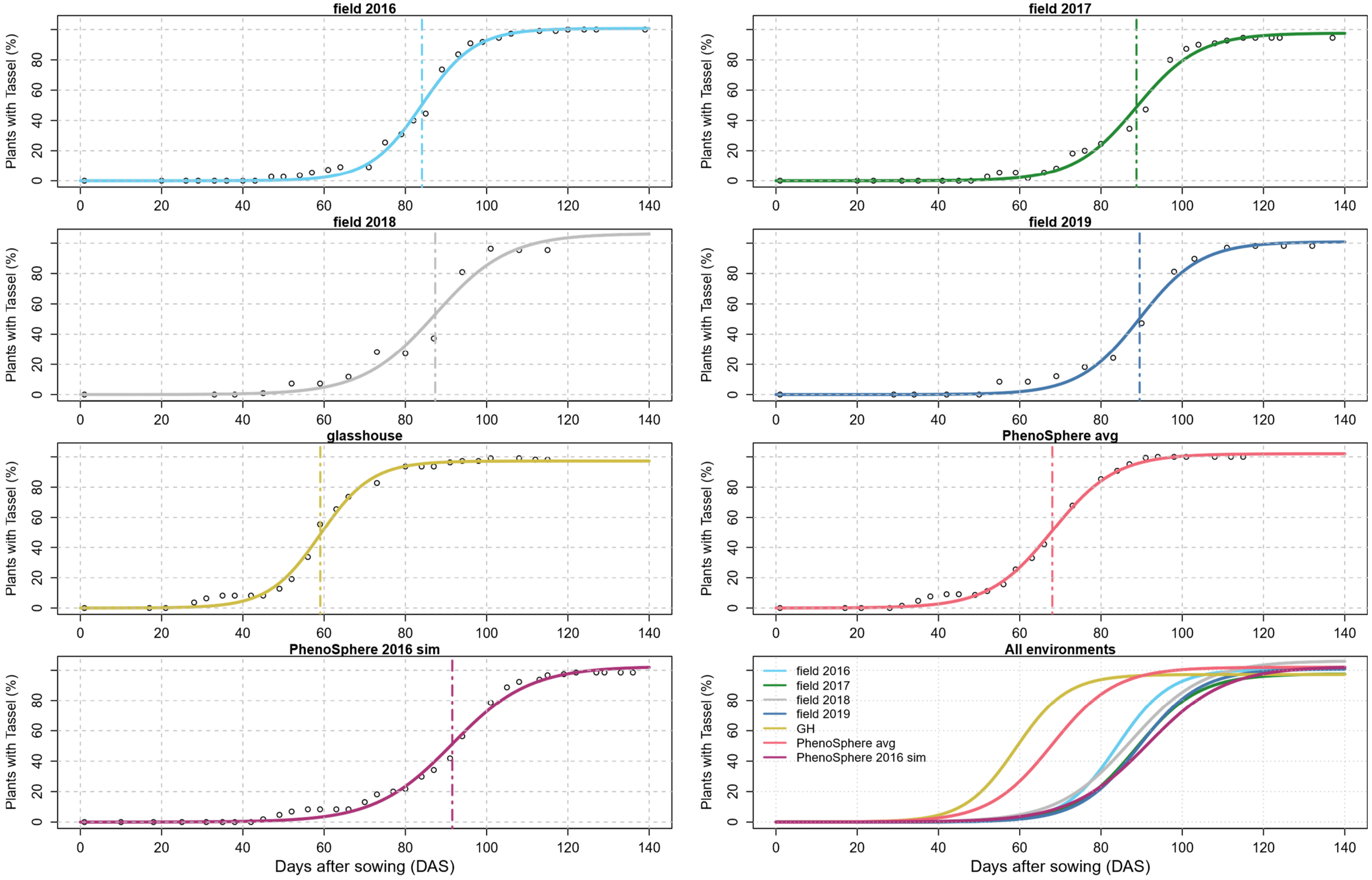

Supplementary Figure 16: **The best fit nonlinear fixed-effect logistic growth model of each environment to the percentage of plants of all genotypes having a tassel over time (days after sowing; DAS).** Vertical dashed lines show the 'xmid' parameter, the days after sowing (DAS), when the inflection point of the logistic growth curve, the rate of plants developing tassels, was maximal. Colors used: field 2016 as Sky Blue (#66CCEE), field 2017 as Forest Green (#228833), field 2018 as Silver (#BBBBBB), field 2019 as San Marino (#4477AA), glasshouse as Turmeric (#CCBB44), PhenoSphere avg as Froly (#EE6677), and PhenoSphere 2016 sim as Royal Heath (#AA3377).

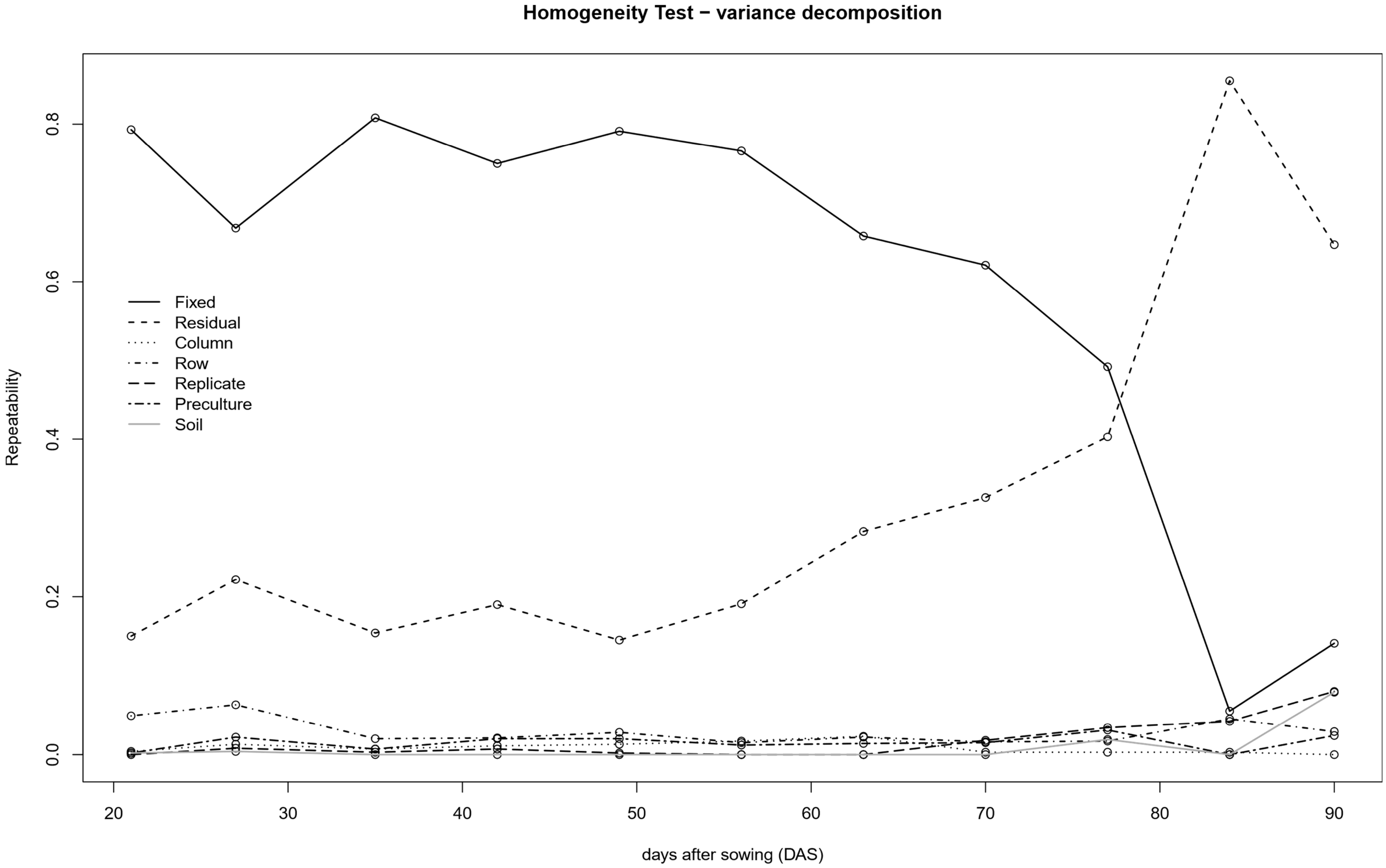

Supplementary Figure 17: Variance decomposition (5) for plant height with the *rptR* package for the homogeneity test experiment in 2019. Repeatability for variance components is plotted against days after sowing (DAS). For statistical parameters see Supplementary Table 2.

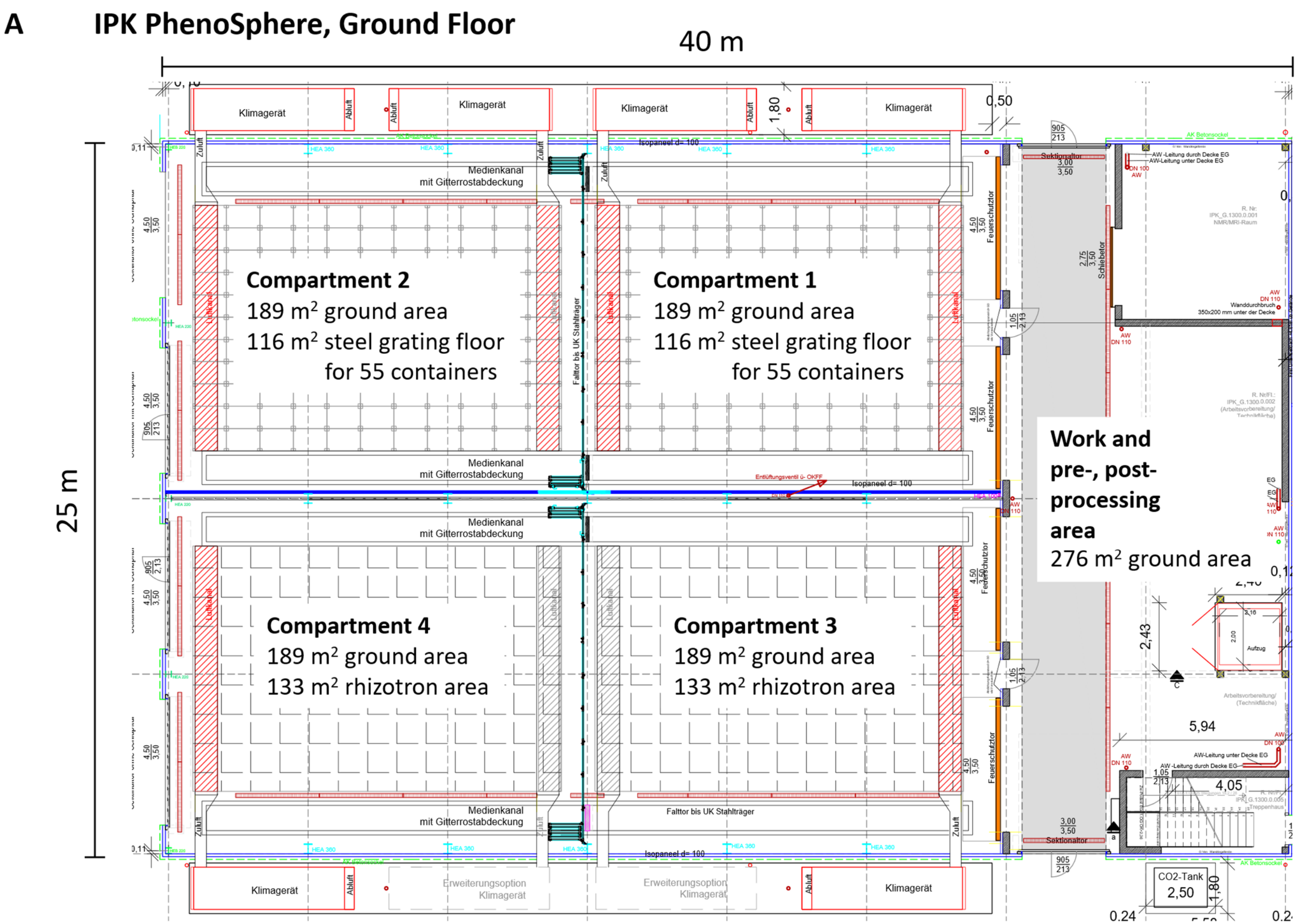

Supplementary Figure 18 A: Ground floor layout of the PhenoSphere. Compartment 1 and 2 contain the container-based cultivation system used here and compartment 3 and 4 house a large rhizotron system for root phenotyping. A large work and pre-/port- processing area is accessible from both systems.

B **IPK PhenoSphere, Compartments 1 & 2**
Container System (top view)

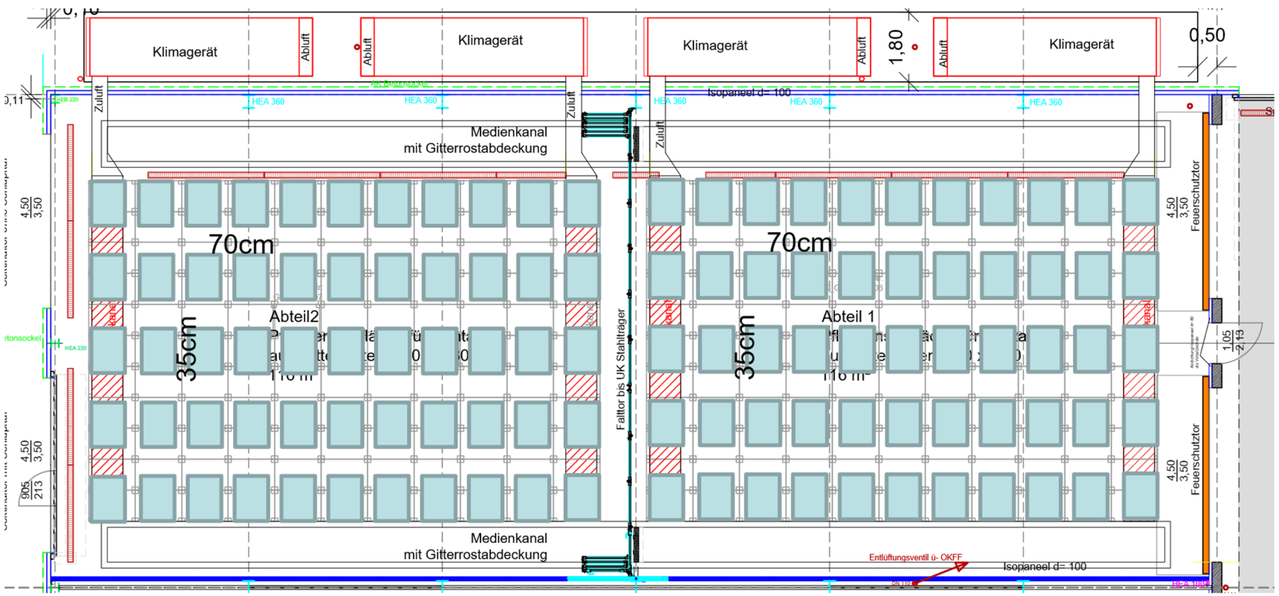

- 5x11 containers per compartment (total110)
- 70/35 cm distances (within/across rows)
- corresponds to the setup of the reference field experiments (2016/17/18/19)

Supplementary Figure 18 B: Maximal container layout. Maximum 55 container layout of compartment 1 and 2 in the container-based cultivation system upon the steel grating floor in the PhenoSphere.

C IPK PhenoSphere, Compartments 1 & 2

Container System (side view)

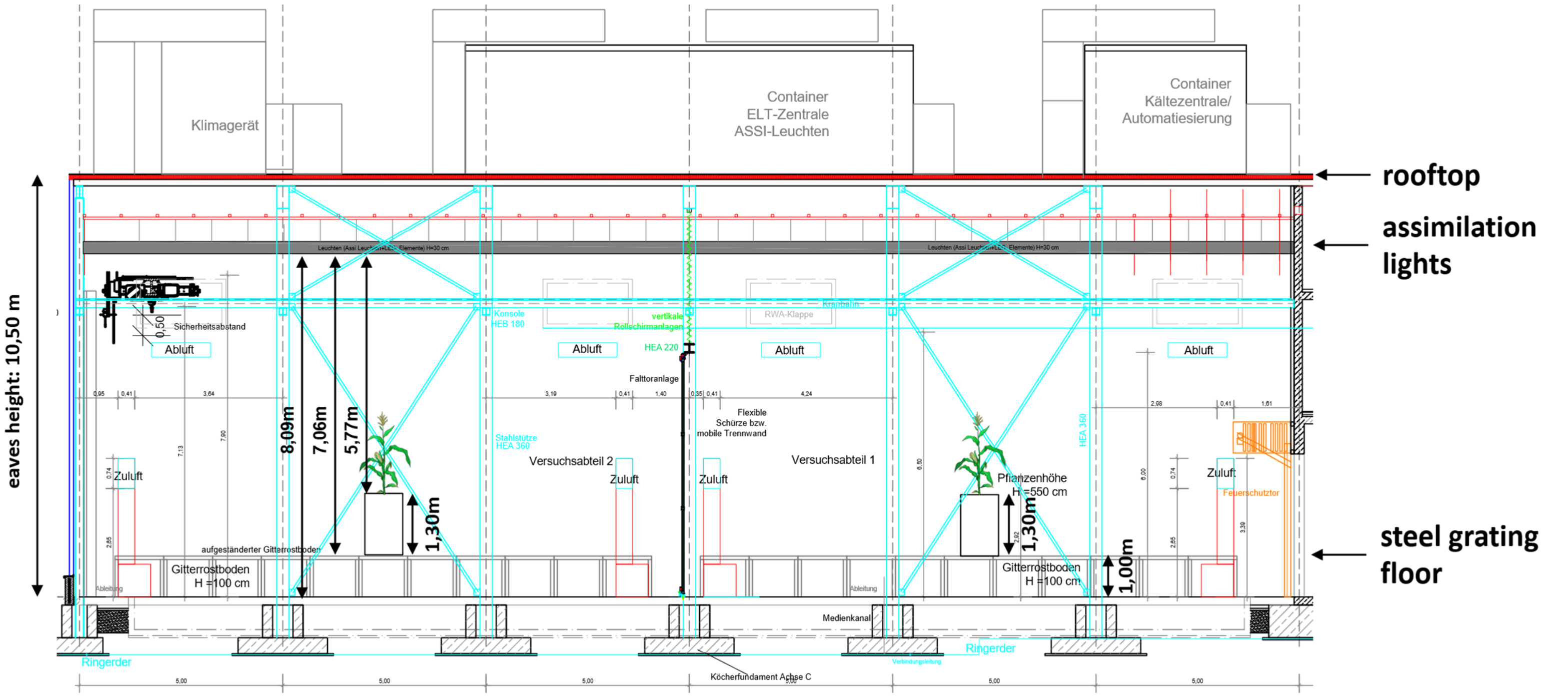

- steel grating floor: 1 m above ground floor (space for air hoses dispensing conditioned fresh air)
- assimilation lights (ceramic metal halide, LEDs, and UV-A bulbs):
  8.09 m above ground floor, 5.77 m above top edge of containers

Supplementary Figure 18 C: Vertical height of compartment 1 and 2. Container (1.30 cm height) are positioned on a steel grating floor (100 cm height). 577 cm air space is available between the container top edge (230 cm) and the assimilation lights (809 cm).

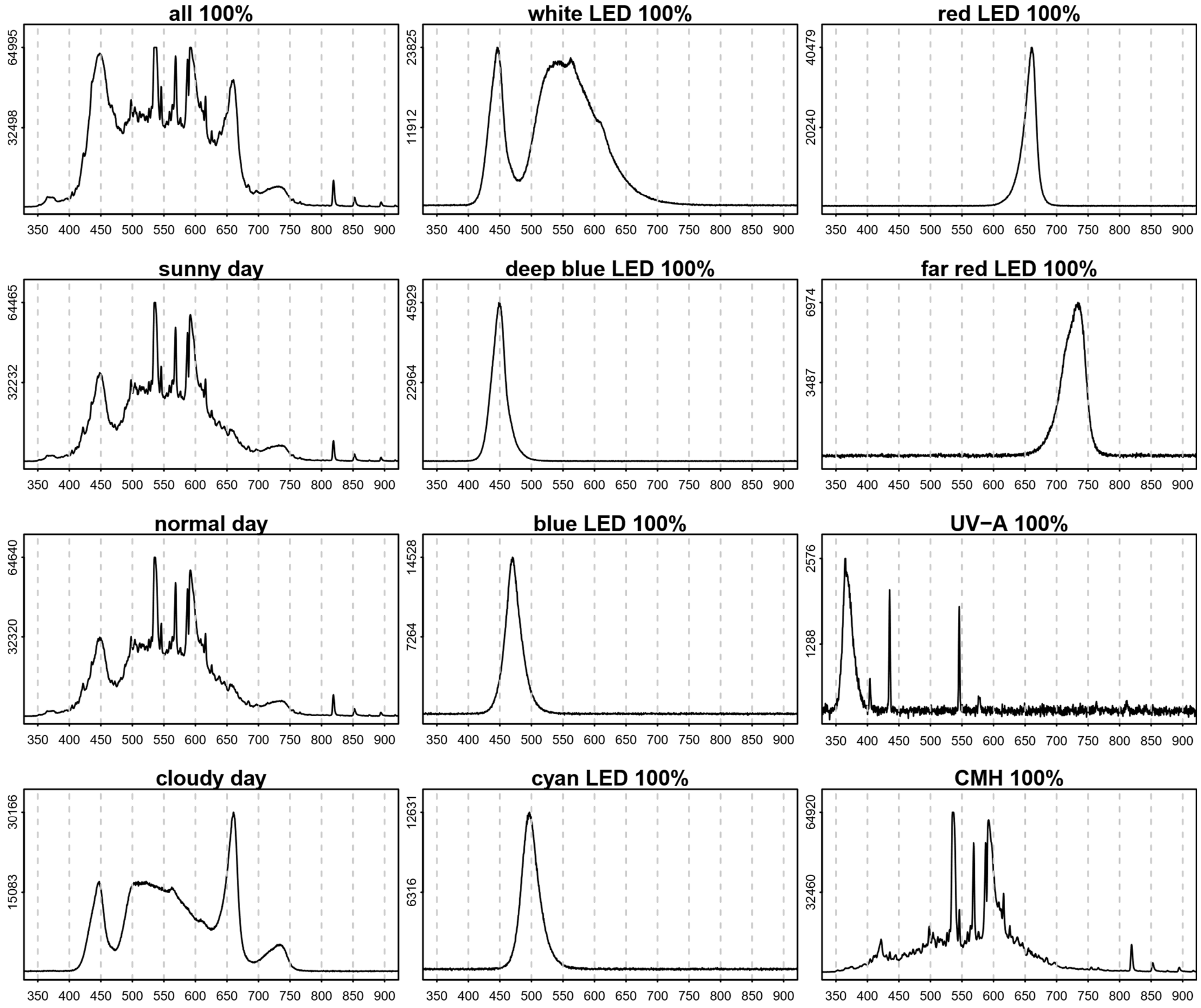

Supplementary Figure 19: Spectra of the PhenoSphere light sources. Spectra (350 nm – 900 nm) of the light sources of the PhenoSphere at 100% intensity individually and combined and in the used combinations for sunny, normal, and cloudy days. Y-axis shows intensity (count) values of the histograms. All spectra were recorded with an Ocean Optics USB2000+XR1-ES (Ocean Insight, D-73760).

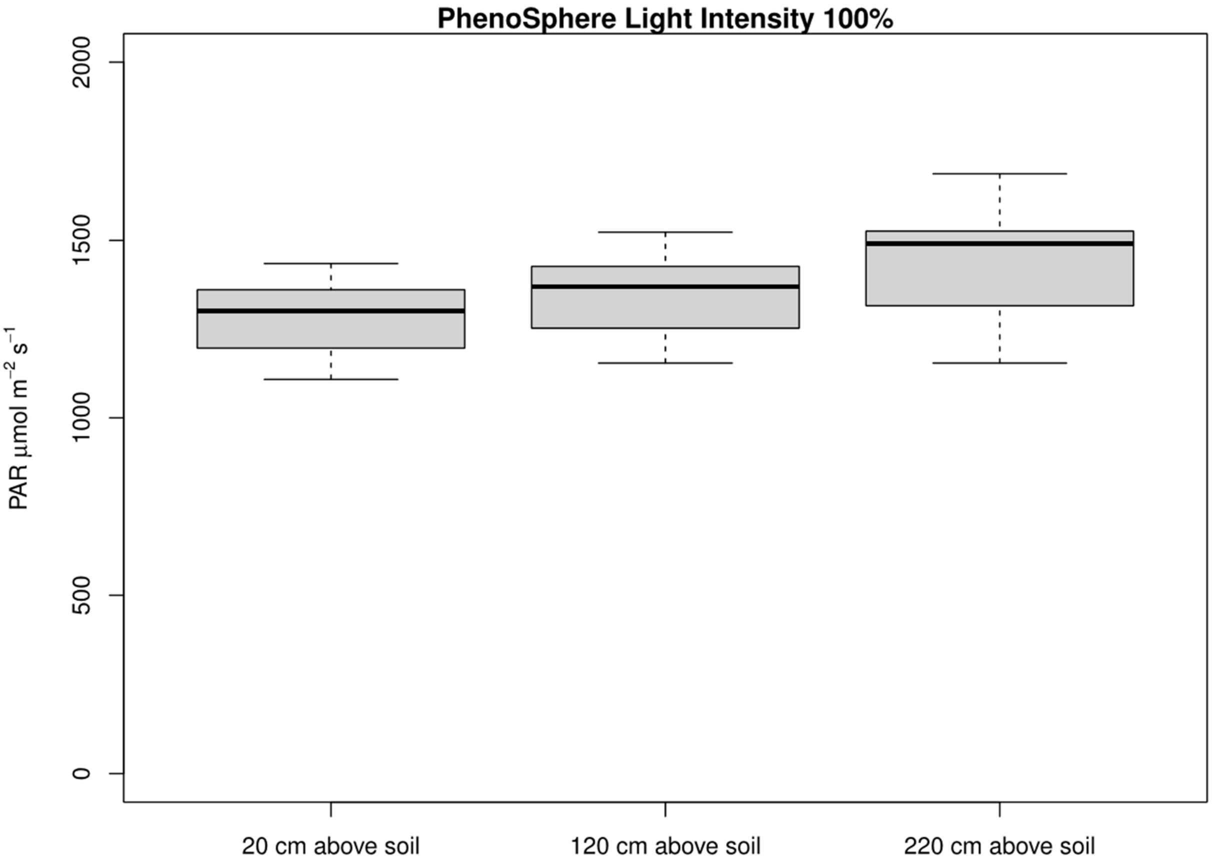

Supplementary Figure 20: Light intensity in the PhenoSphere at different heights. Light intensity (µmol m^{-2} s^{-1}) of all light source at 100 % intensity (spectrum see Supplementary Fig. 19) at 20 cm (average 1277 µmol m^{-2} s^{-1}), 120 cm (average 1348 µmol m^{-2} s^{-1}), and 220 cm (average 1436 µmol m^{-2} s^{-1}) above soil level measured with a Licor LI-250A light meter coupled with a LI-190R-BNC-2 quantum sensor (LI-COR Bioscience GmbH, D-61352). The light intensities were measured at 13 evenly spaced positions at specified heights. The boxplots show the median, the box is the interquartile range, and the whiskers range to 1.5 x interquartile range.

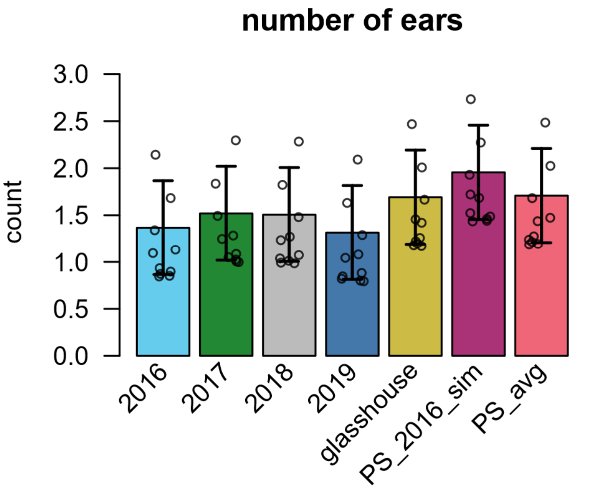

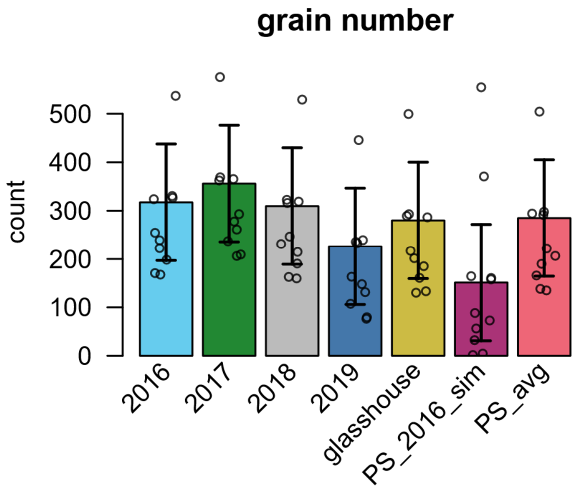

number of ears	2017	2018	2019	glasshouse	PS_2016_sim	PS_avg
2016	0.94	0.96	1.00	0.31	0.00	0.25
2017		1.00	0.81	0.90	0.06	0.86
2018			0.85	0.87	0.05	0.81
2019				0.16	0.00	0.12
glasshouse					0.56	1.00
PS_2016_sim						0.63

grain number	2017	2018	2019	glasshouse	PS_2016_sim	PS_avg
2016	0.97	1.00	0.36	0.98	0.01	0.99
2017		0.93	0.06	0.58	0.00	0.65
2018			0.47	0.99	0.01	1.00
2019				0.88	0.60	0.82
glasshouse					0.06	1.00
PS_2016_sim						0.05

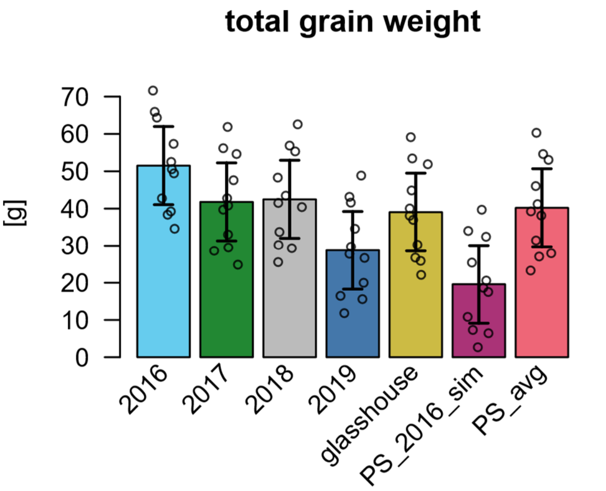

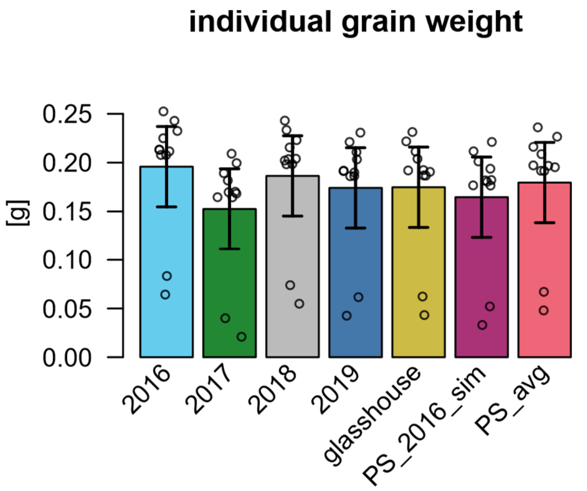

total grain weight	2017	2018	2019	glasshouse	PS_2016_sim	PS_avg
2016	0.52	0.60	0.00	0.22	0.00	0.33
2017		1.00	0.18	1.00	0.00	1.00
2018			0.14	0.99	0.00	1.00
2019				0.45	0.59	0.32
glasshouse					0.01	1.00
PS_2016_sim						0.00

Individual grain weight	2017	2018	2019	glasshouse	PS_2016_sim	PS_avg
2016	0.00	0.98	0.43	0.47	0.08	0.75
2017		0.04	0.43	0.40	0.92	0.18
2018			0.92	0.93	0.42	1.00
2019				1.00	0.97	1.00
glasshouse					0.96	1.00
PS_2016_sim						0.81

Supplementary Figure 21: Yield of the maize population in all environments. Yield components are plotted as fixed effects for each environment together with the 95% confidence interval (error bar) calculated from the genotypes as a random effect based on the BLUPs of the raw values. Dot plots show the fitted values of the model. Number of ears before harvest of the ears; grain number gives the total number of grains per plant; total grain weight gives the sum of the weight (in gram) of all grains per plant; average individual grain weight (in gram) is a calculated parameter derived from the grain number and the total grain weight. Tables show Tukey-adjusted pairwise p-values. Colors used: field 2016 as Sky Blue (#66CCEE), field 2017 as Forest Green (#228833), field 2018 as Silver (#BBBBBB), field 2019 as San Marino (#4477AA), glasshouse as Turmeric (#CCBB44), PhenoSphere avg as Froly (#EE6677), and PhenoSphere 2016 sim as Royal Heath (#AA3377).

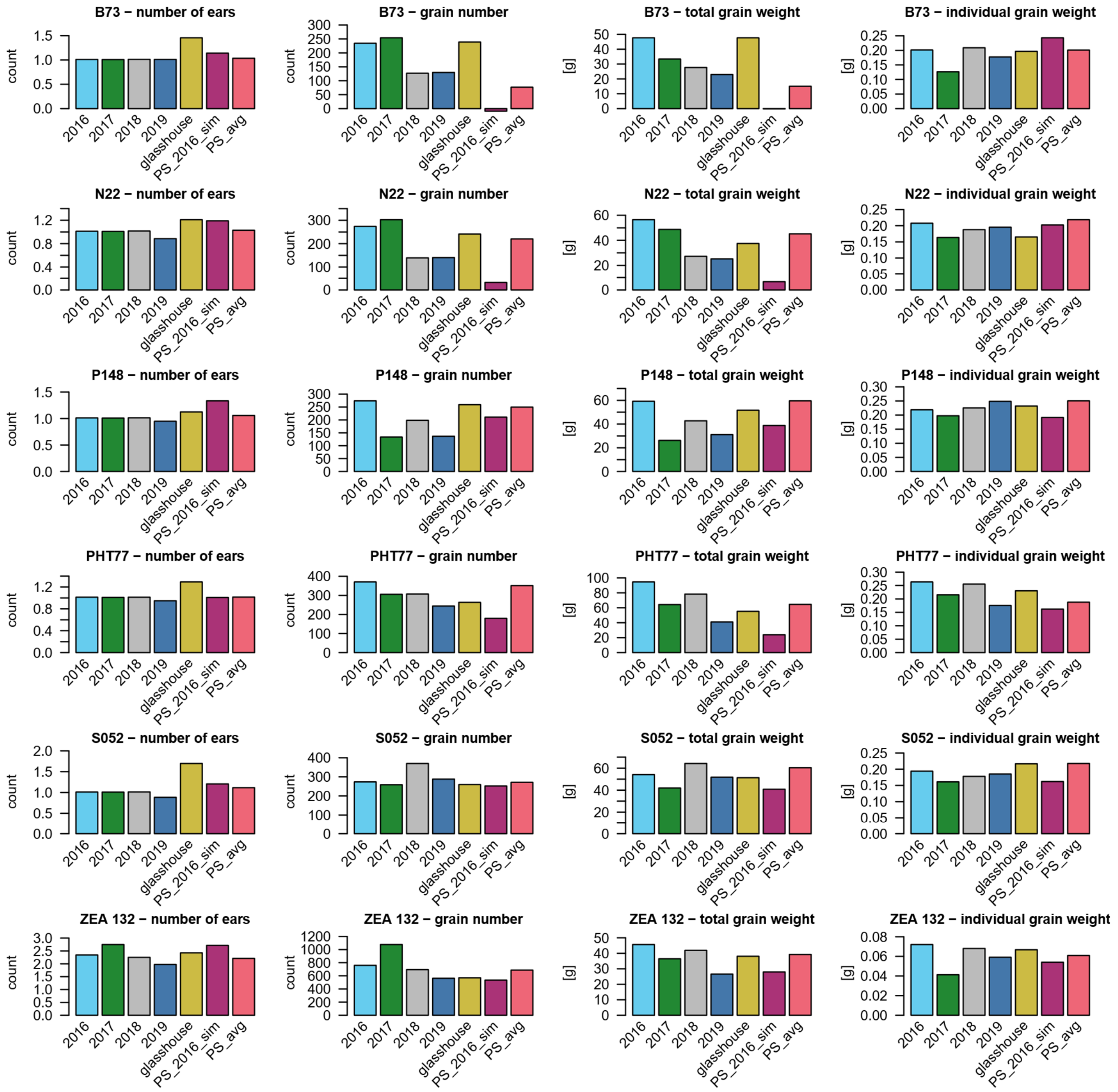

Supplementary Figure 22 part 1: Yield of single genotypes in all environments. Yield components are plotted as the BLUPs for each environment from the raw values individually for each genotype. Number of ears before harvest of the ears; grain number gives the total number of grains per plant; total grain weight gives the sum of the weight (in gram) of all grains per plant; average individual grain weight (in gram) is a calculated parameter derived from the grain number and the total grain weight. Colors used: field 2016 as Sky Blue (#66CCEE), field 2017 as Forest Green (#228833), field 2018 as Silver (#BBBBBB), field 2019 as San Marino (#4477AA), glasshouse as Turmeric (#CCBB44), PhenoSphere avg as Froly (#EE6677), and PhenoSphere 2016 sim as Royal Heath (#AA3377).

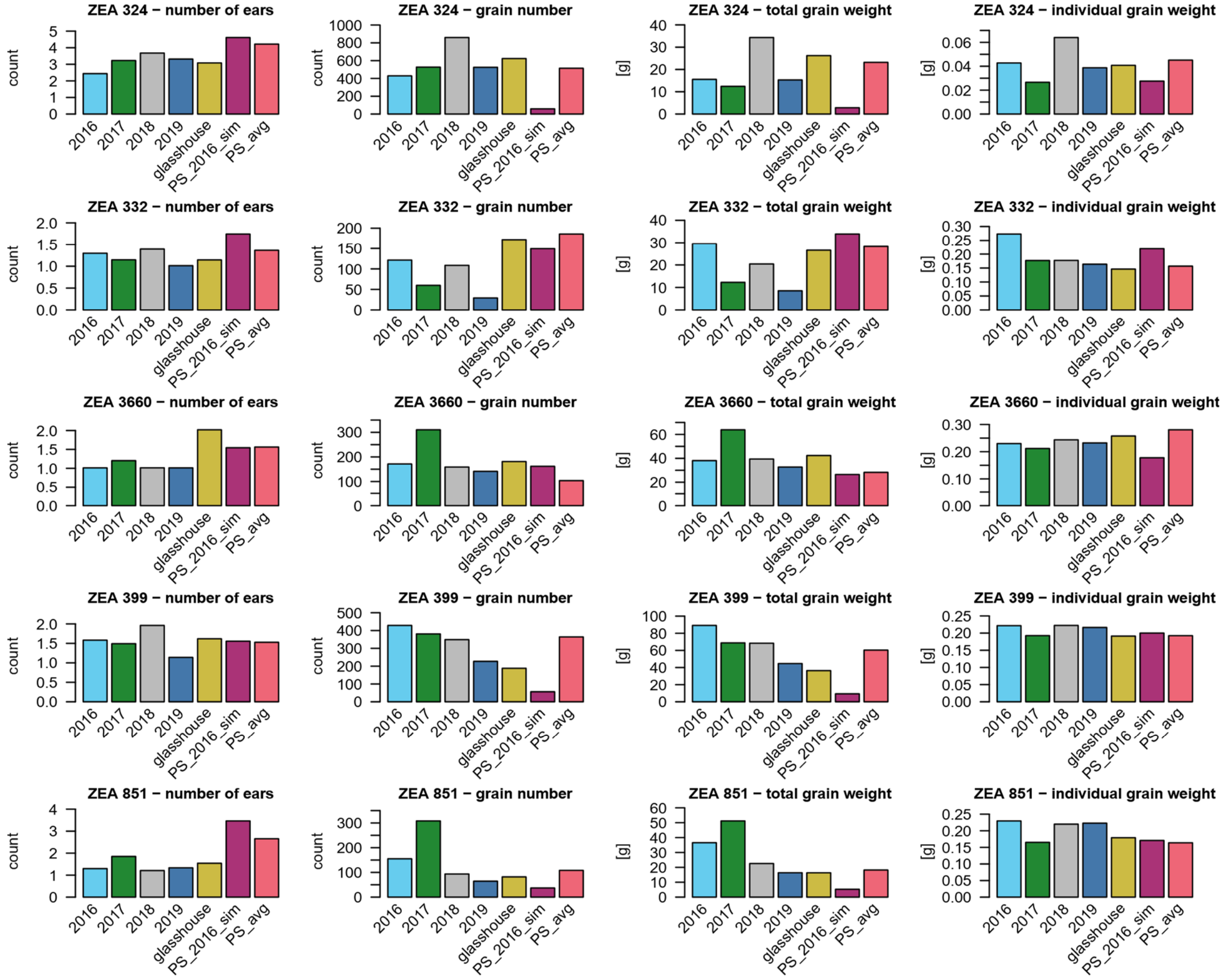

Supplementary Figure 22 part 2: Yield of single genotypes in all environments. Yield components are plotted as the BLUPs for each environment from the raw values individually for each genotype. Number of ears before harvest of the ears; grain number gives the total number of grains per plant; total grain weight gives the sum of the weight (in gram) of all grains per plant; average individual grain weight (in gram) is a calculated parameter derived from the grain number and the total grain weight. Colors used: field 2016 as Sky Blue (#66CCEE), field 2017 as Forest Green (#228833), field 2018 as Silver (#BBBBBB), field 2019 as San Marino (#4477AA), glasshouse as Turmeric (#CCBB44), PhenoSphere avg as Froly (#EE6677), and PhenoSphere 2016 sim as Royal Heath (#AA3377).

Supplementary Table 1: *Zea mays* population of the 11 inbred lines with origins from North America, Europe, and Asia. The lines abbreviated with 'ZEA' were supplied by the IPK Genbank and are accessible via their digital object identifier (DOI).

Accession	Taxa	Year	Donor/Breeder	Origin	Background	DOI
B73	*Zea mays* ssp. *mays*	1972	Iowa Agric & Home Econ Exp Stn	United States, Iowa	Iowa Stiff Stalk Synthetic C5	
N22	*Zea mays* ssp. *mays*	unknown	unknown	United States, Nebraska	Krug Yellow Dent	
P148	*Zea mays* ssp. *mays*	unknown	unknown	Germany	Non-Stiff Stalk	
PHT77	*Zea mays* ssp. *mays*	1988	Pioneer Hi-Bred International, Inc.	United States, Minnesota	Non-Stiff Stalk Synthetic	
S052	*Zea mays* ssp. *mays*	unknown	unknown	Germany	Non-Stiff Stalk	
ZEA 132	*Zea mays* L. subsp. *everta* (Sturtev.) Zhuk. var. *glaucornis* Alef.	1953	Genetikai Osztaly Budapest	unknown	advanced/improved cultivar	doi.org/10.25642/IPK/GBIS/33630
ZEA 324	*Zea mays* L. subsp. *everta* (Sturtev.) Zhuk. var. *haematornis* Alef.	1968	Agricultural Botanical Garden Bucharest	unknown	advanced/improved cultivar	doi.org/10.25642/IPK/GBIS/33799
ZEA 332	*Zea mays* L. subsp. *indurata* (Sturtev.) Zhuk. var. *vulgata* Körn.	1967	VIR Leningrad	Soviet Union	advanced/improved cultivar	doi.org/10.25642/IPK/GBIS/33807
ZEA 3660	*Zea mays* L. subsp. *indentata* (Sturtev.) Zhuk. var. *flavorubra* Körn.	2003	BAZ, Braunschweig Genetic Resources Centre	China, Jilin	Breeding/research material	doi.org/10.25642/IPK/GBIS/234225
ZEA 399	*Zea mays* L.	1991	National Institute of Agrobiological Resources Tsukuba	Democratic People's Republic of Korea (DPRK)	Breeding/research material	doi.org/10.25642/IPK/GBIS/70927
ZEA 851	*Zea mays* L. subsp. indurata (Sturtev.) Zhuk.	1991	National Institute of Agrobiological Resources Tsukuba	Democratic People's Republic of Korea (DPRK)	Breeding/research material	doi.org/10.25642/IPK/GBIS/70928

Supplementary Table 2: Root mean squared error (RMSE). RMSE of the hourly raw temperature profiles, the hourly vapor pressure deficit (VPD) profiles, and the thermal time days of the field 2016 environment (treated as template) against the other environments (treated as predicted).

RMSE vs field 2016	temperature	VPD	thermal time days
field 2017	5.63	0.79	0.39
field 2018	5.78	0.88	0.4
field 2019	5.27	0.75	0.35
glasshouse	6.93	1.3	0.53
PhenoSphere avg	4.94	0.65	0.34
PhenoSphere 2016 sim	3.06	0.41	0.17

Supplementary Table 3: Contribution of technical factors to the phenotypic variance. Repeatability at all days after sowing (DAS) of the homogeneity experiment and the statistical parameters from the variance decomposition. P-values were estimated from 1000 bootstrapping cycles for the random effects using a likelihood-ratio test. No p-values can be estimated for the fixed effect and the residuals. Factors were considered to contribute significantly when the p-value was lower than 0.05 and the confidence intervals did not contain the zero.

Factor	Repeatability	P.value	CI.2.5%	CI.97.5%	DAS
Row	0.049	0	0.004509	0.12916	21
Column	0.004	0.091	0	0.013697	21
Soil	0.002	0.266	0	0.012284	21
Preculture	0.002	0.273	0	0.01094	21
Replicate	0	1	0	0.006069	21
Residual	0.15	NA	0.12498	0.17638	21
Fixed	0.793	NA	0.726124	0.841356	21
Row	0.063	0	0.004805	0.169253	27
Column	0.013	0.04	0	0.038554	27
Soil	0.004	0.272	0	0.034111	27
Preculture	0.022	0	0	0.052494	27
Replicate	0.008	0.139	0	0.036238	27
Residual	0.222	NA	0.182167	0.262448	27
Fixed	0.668	NA	0.586741	0.732424	27
Row	0.02	0	0	0.055898	35
Column	0.007	0.038	0	0.022393	35
Soil	0	1	0	0.01068	35
Preculture	0.007	0.028	0	0.021023	35
Replicate	0.003	0.216	0	0.016277	35
Residual	0.154	NA	0.131003	0.182216	35
Fixed	0.808	NA	0.765873	0.841774	35
Row	0.021	0	0	0.061917	42
Column	0.011	0.042	0	0.034226	42
Soil	0	1	0	0.01612	42
Preculture	0.02	0	0	0.049545	42
Replicate	0.007	0.196	0	0.031343	42
Residual	0.19	NA	0.158614	0.221926	42
Fixed	0.75	NA	0.699756	0.791802	42
Row	0.028	0	0.001129	0.078389	49
Column	0.013	0.002	0	0.034782	49
Soil	0	1	0	0	49
Preculture	0.02	0	0.002205	0.045453	49
Replicate	0.002	0.277	0	0.019124	49
Residual	0.145	NA	0.122447	0.172201	49
Fixed	0.791	NA	0.740323	0.830177	49
Row	0.015	0.001	0	0.044987	56
Column	0.017	0.001	0	0.038022	56
Soil	0	1	0	0.01417	56
Preculture	0.012	0.007	0	0.031587	56
Replicate	0	0.5	0	0.015774	56
Residual	0.191	NA	0.162714	0.220004	56
Fixed	0.766	NA	0.722625	0.80116	56
Row	0.022	0.001	0	0.067884	63
Column	0.023	0.004	0	0.055013	63
Soil	0	1	0	0.019362	63
Preculture	0.014	0.024	0	0.041584	63
Replicate	0	1	0	0.023967	63
Residual	0.283	NA	0.235031	0.326904	63
Fixed	0.658	NA	0.596858	0.708648	63
Row	0.016	0.007	0	0.055639	70
Column	0.003	0.328	0	0.025562	70
Soil	0	0.5	0	0.019912	70
Preculture	0.015	0.025	0	0.040096	70
Replicate	0.018	0.044	0	0.05702	70
Residual	0.326	NA	0.280462	0.375146	70
Fixed	0.621	NA	0.560034	0.672844	70

Row	0.017	0.015	0	0.060067	77
Column	0.003	0.36	0	0.035331	77
Soil	0.019	0.216	0	0.098403	77
Preculture	0.031	0.003	0	0.077041	77
Replicate	0.034	0.015	0	0.097175	77
Residual	0.403	NA	0.338842	0.472281	77
Fixed	0.492	NA	0.41035	0.56192	77
Row	0.045	0.004	0	0.131564	84
Column	0.003	0.425	0	0.050788	84
Soil	0	1	0	0.027221	84
Preculture	0	1	0	0	84
Replicate	0.042	0.057	0	0.131432	84
Residual	0.855	NA	0.734333	0.93816	84
Fixed	0.055	NA	0.018873	0.107124	84
Row	0.029	0.008	0	0.096457	90
Column	0	0.5	0	0	90
Soil	0.079	0.034	0	0.26965	90
Preculture	0.024	0.051	0	0.074704	90
Replicate	0.08	0.014	0.001089	0.207843	90
Residual	0.647	NA	0.497429	0.782657	90
Fixed	0.141	NA	0.081694	0.221062	90

Supplementary Table 4: Illumination program of the PhenoSphere at a *normal day*. Values for each lighting channel are given in percent of maximum. C stands for constant and R for a linear ramping. LED light channels (W, white; DB, dark blue; R, red; FR, far red), UV, and 4 channels of equal intensity of metal halide lamps. Sin symbolizes an oscillation between the two values following a sinus curve. LED-B (blue) was turned off. Sunrise and sunset have been adjusted weekly.

Time	LED-W	LED-DB	LED-CB	LED-R	LED-FR	UV(A)	CMH-Gr.1	CMH-Gr.2	CMH-Gr.3	CMH-Gr.4
**0:00**	0	0	0	0	0	0	0	0	0	0
	C		C	C	C	C				
**6:00**	0		0	0	0	0				
	R		R	R	R	R	C			
**7:00**	50		50	50	50	15	100			
	R						C			
**7:03**	10		10	10	10		100			
	R		R	R	R	R	C			
**8:00**	50		50	50	50	30	100			
	Sin_50-100		Sin_50-100	Sin_50-100	Sin_50-100	R	C	C		
**9:00**	50		50	50	50	50	100	100		
	R		R	R	R		C	C		
**9:03**	30		30	30	30					
	R		R	R	R	R	C	C	C	
**10:00**	70		70	70	70	60	100	100	100	
	R		R	R	R					
**10:03**	20		20	20	20					
	Sin_10-50	C	Sin_10-50	Sin_10-50	Sin_10-50	R	C	C	C	
**11:00**	50	0	50	50	50	70	100	100	100	
	R	R	R	R	R	R				
**12:00**	75	15	75	8	75	75				
	C	C	C	C	C	C	C	C	C	C
**13:00**	75	15	75	8	75	75	0	100	100	100
	Sin_10-75	Sin_2-15	Sin_10-75	Sin_1-8	Sin_10-75	C	C	C	C	C
**14:00**	75	15	75	8	75	75	0	100	100	100
	C	C	C	C	C	C	C	C	C	C
**15:00**	75	15	75	8	75	75	0	100	100	100
	R	R	R	R	R	R	C	C	C	C
**16:00**	20	0	20	20	20	70	0	100	100	100
	Sin_10-50	C	Sin_10-50	Sin_10-50	Sin_10-50	R		C	C	C
**17:00**	70		70	70	70	60		0	100	100
	R		R	R	R	R			C	C
**18:00**	30		30	30	30	50			0	100
	Sin_50-100		Sin_50-100	Sin_50-100	Sin_50-100	R				C
**19:00**	50		50	50	50	30				100
	R		R	R	R	R				
**20:00**	10		10	10	10	15				C
**20:00**	50		50	50	50	15				0
	R		R	R	R	R				
**21:00**	0		0	0	0	0				
	C	C	C	C	C	C	C	C	C	C
**0:00**	0	0	0	0	0	0	0	0	0	0

Supplementary Table 5: Illumination of the PhenoSphere at a *sunny day*. Values for each lighting channel are given in percent of maximum. C stands for constant and R for a linear ramping. LED light channels (W, white; DB, dark blue; R, red; FR, far red), UV, and 4 channels of equal intensity of metal halide lamps. Sin symbolizes an oscillation between the two values following a sinus curve. LED-B (blue) was turned off. Sunrise and sunset have been adjusted weekly.

Time	LED-W	LED-DB	LED-CB	LED-R	LED-FR	UV(A)	CMH-Gr.1	CMH-Gr.2	CMH-Gr.3	CMH-Gr.4
**0:00**	0	0	0	0	0	0	0	0	0	0
	C		C	C	C	C				
**6:00**	0		0	0	0	0				
	R		R	R	R	R	C			
**7:00**	50		50	50	50	15	100			
	R						C			
**7:03**	10		10	10	10		100			
	R		R	R	R	R	C			
**8:00**	50		50	50	50	30	100			
	Sin_50-90		Sin_50-90	Sin_50-90	Sin_50-90	R	Sin_80-100	C		
**9:00**	100		100	100	100	50	100	100		
	R		R	R	R		C	C		
**9:03**	30		30	30	30					
	R		R	R	R	R	C	C	C	
**10:00**	70		70	70	70	60	100	100	100	
	R		R	R	R					
**10:03**	20		20	20	20					
	R	C	R	R	R	R	C	C	C	
**11:00**	50	0	50	50	50	70	100	100	100	
	R	R	R	R	R	R				
**12:00**	80	20	80	8	80	80				
	C	C	C	C	C	C	C	C	C	C
**13:00**	100	20	100	8	100	80	0	100	100	100
**13:03**	80		80		80	80				
	C	C	C	C	C	C	C	C	C	C
**14:00**	80	20	80	8	80	80	0	100	100	100
	C	R	R	R	R	R	C	C	C	C
**15:00**	80	15	75	8	75	75	0	100	100	100
	R	R	R	R	R	R	C	C	C	C
**16:00**	30	0	30	30	30	70	0	100	100	100
	Sin_20-50	C	Sin_20-50	Sin_20-50	Sin_20-50	R		Sin_80-100	Sin_80-100	Sin_80-100
**17:00**	70		70	70	70	60		0	100	100
	R		R	R	R	R			C	C
**18:00**	30		30	30	30	50			0	100
**18:00**	100		100	100	100	R				C
	R		R	R	R					
**19:00**	50		50	50	50	30				100
	R		R	R	R	R				
**20:00**	10		10	10	10	15				C
**20:00**	50		50	50	50	15				0
	R		R	R	R	R				
**21:00**	0		0	0	0	0				
	C	C	C	C	C	C	C	C	C	C
**0:00**	0	0	0	0	0	0	0	0	0	0

Supplementary Table 6: Illumination of the PhenoSphere at a *cloudy day*. Values for each lighting channel are given in percent of maximum. C stands for constant and R for a linear ramping. LED light channels (W, white; DB, dark blue; R, red; FR, far red), UV, and 4 channels of equal intensity of metal halide lamps. Sin symbolizes an oscillation between the two values following a sinus curve. LED-B (blue) was turned off. Sunrise and sunset have been adjusted weekly.

Time	LED-W	LED-DB	LED-CB	LED-R	LED-FR	UV(A)	CMH-Gr.1	CMH-Gr.2	CMH-Gr.3	CMH-Gr.4
**0:00**	0	0	0	0	0	0	0	0	0	0
	C		C	C	C	C				
**6:00**	0		0	0	0	0				
	R		R	R	R	R				
**7:00**	15		15	15	15	6				
	Sin_10_30		Sin_10_30	Sin_10_30	Sin_10_30					
**8:00**										
	Sin_20_50		Sin_20_50	Sin_20_50	Sin_20_50	R				
**9:00**	45		45	45	45	15				
	R		R	R	R	R				
**10:00**	55		55	55	55	20				
	Sin_45_70		Sin_45_70	Sin_45_70	Sin_45_70	R				
**11:00**						25				
	Sin_55_75		Sin_55_75	Sin_55_75	Sin_55_75	R				
**12:00**						25				
**13:00**	65		65	65	65	25				
	R		R	R	R	R				
**14:00**	55		55	55	55	20				
	Sin_60_40		Sin_60_40	Sin_60_40	Sin_60_40	R				
**15:00**	45		45	45	45	15				
	R		R	R	R	R				
**16:00**	35		35	35	35	10				
	Sin_40_25		Sin_40_25	Sin_40_25	Sin_40_25	R				
**17:00**	30		30	30	30	8				
	R		R	R	R	R				
**18:00**	25		25	25	25	7				
	Sin_30_20		Sin_30_20	Sin_30_20	Sin_30_20	R				
**19:00**						6				
	Sin_25_10		Sin_25_10	Sin_25_10	Sin_25_10	C				
**20:00**	15		15	15	15	6				
	R		R	R	R	R				
**21:00**	0		0	0	0	0	0	0	0	0
	C	C	C	C	C	C	C	C	C	C
**0:00**	0	0	0	0	0	0	0	0	0	0
