# Supplementary material for: Natural plant growth and development achieved in the IPK PhenoSphere by dynamic environment simulation
Source: Nat Commun. 2023 Sep 18;14:5783. doi: 10.1038/s41467-023-41332-4 (PMC10507097; doi:10.1038/s41467-023-41332-4)
Supplement: Supplementary file 2 — Description of Additional Supplementary Files [file 41467_2023_41332_MOESM2_ESM.pdf]

Title: Supplementary Data 1:
Description: Sensor data from the climate in each cultivation. The file provides the sensor data in an hour resolution for field 2016 – 2019 and the glasshouse and in a sub-hour resolution for the PhenoSphere avg and 2016 sim.

Title: Supplementary Data 2:
Description: Phenotypic Data of all performed cultivations. The file provides the vegetative and yield phenotypic data of all cultivations.

Title: Supplementary Data 3:
Description: Statistic of the estimated means comparison of the nonlinear model parameters between each cultivation experiment. The file provides comparisons of the “Asym”, “A”, “B”, “xmid”, and “scal” parameters calculated from the nonlinear model fitted to the corresponding phenotypic data.

Title: Supplementary Code 1:
Description: R scripts and required data to reproduce figures and statics. The folder contains a readme, which refers to the file paths and scripts of each figure and supplementary figure.
