# Supplementary material for: Natural plant growth and development achieved in the IPK PhenoSphere by dynamic environment simulation
Source: Nat Commun. 2023 Sep 18;14:5783. doi: 10.1038/s41467-023-41332-4 (PMC10507097; doi:10.1038/s41467-023-41332-4)
Supplement: Supplementary file 3 — Reporting Summary [file 41467_2023_41332_MOESM3_ESM.pdf]

nature portfolio

Corresponding author(s): Marc Christian Heuermann

Last updated by author(s): Aug 28, 2023

Reporting Summary

Nature Portfolio wishes to improve the reproducibility of the work that we publish. This form provides structure for consistency and transparency in reporting. For further information on Nature Portfolio policies, see our Editorial Policies and the Editorial Policy Checklist.

Statistics

For all statistical analyses, confirm that the following items are present in the figure legend, table legend, main text, or Methods section.

n/a	Confirmed	
☐	☒	The exact sample size (*n*) for each experimental group/condition, given as a discrete number and unit of measurement
☐	☒	A statement on whether measurements were taken from distinct samples or whether the same sample was measured repeatedly
☐	☒	The statistical test(s) used AND whether they are one- or two-sided<br>*Only common tests should be described solely by name; describe more complex techniques in the Methods section.*
☐	☒	A description of all covariates tested
☐	☒	A description of any assumptions or corrections, such as tests of normality and adjustment for multiple comparisons
☐	☒	A full description of the statistical parameters including central tendency (e.g. means) or other basic estimates (e.g. regression coefficient) AND variation (e.g. standard deviation) or associated estimates of uncertainty (e.g. confidence intervals)
☐	☒	For null hypothesis testing, the test statistic (e.g. *F*, *t*, *r*) with confidence intervals, effect sizes, degrees of freedom and *P* value noted<br>*Give P values as exact values whenever suitable.*
☒	☐	For Bayesian analysis, information on the choice of priors and Markov chain Monte Carlo settings
☐	☒	For hierarchical and complex designs, identification of the appropriate level for tests and full reporting of outcomes
☐	☒	Estimates of effect sizes (e.g. Cohen's *d*, Pearson's *r*), indicating how they were calculated

Our web collection on statistics for biologists contains articles on many of the points above.

Software and code

Policy information about availability of computer code

Data collection	Data was collected as manual measurement directly on the maize plant and sensor data was collected from the IPK weather station and sensors inside the indoor environments. Data was collected in Microsoft Excel and saved as a Comma Separated Values file.
Data analysis	All data analsysis was performed with R version 4.1.0 and following packages, nlme package version 3.1-152, lme4 package version 1.1.27.1, forecast package version 8.15, gplots package version 3.1.1, emmeans package version 1.6.2-1, bbmle package version 1.0.24, MuMIn package version 1.43.17, rptR package version 0.9.22

For manuscripts utilizing custom algorithms or software that are central to the research but not yet described in published literature, software must be made available to editors and reviewers. We strongly encourage code deposition in a community repository (e.g. GitHub). See the Nature Portfolio guidelines for submitting code & software for further information.

Data

Policy information about availability of data

All manuscripts must include a data availability statement. This statement should provide the following information, where applicable:

- Accession codes, unique identifiers, or web links for publicly available datasets
- A description of any restrictions on data availability
- For clinical datasets or third party data, please ensure that the statement adheres to our policy

The authors declare that sensor and phenotypic data from each environment generated in this study are provided in Supplementary Data 1 and 2, respectively. The

processed data are provided within the Supplementary Code 1 folder. Six maize accession (ZEA 132 doi.org/10.25642/IPK/GBIS/33630, ZEA 324 doi.org/10.25642/IPK/GBIS/33799, ZEA 332 doi.org/10.25642/IPK/GBIS/33807, ZEA 3660 doi.org/10.25642/IPK/GBIS/234225, ZEA 399 doi.org/10.25642/IPK/GBIS/70927, ZEA 851 doi.org/10.25642/IPK/GBIS/70928) were sourced from the IPK Gene Bank (https://gbis.ipk-gatersleben.de/gbis2i/).

Research involving human participants, their data, or biological material

Policy information about studies with human participants or human data. See also policy information about sex, gender (identity/presentation), and sexual orientation and race, ethnicity and racism.

Reporting on sex and gender	Not Applicable
Reporting on race, ethnicity, or other socially relevant groupings	Not Applicable
Population characteristics	Not Applicable
Recruitment	Not Applicable
Ethics oversight	Not Applicable

Note that full information on the approval of the study protocol must also be provided in the manuscript.

Field-specific reporting

Please select the one below that is the best fit for your research. If you are not sure, read the appropriate sections before making your selection.

☐ Life sciences ☐ Behavioural & social sciences ☒ Ecological, evolutionary & environmental sciences

For a reference copy of the document with all sections, see nature.com/documents/nr-reporting-summary-flat.pdf

Ecological, evolutionary & environmental sciences study design

All studies must disclose on these points even when the disclosure is negative.

Study description	A Zea mays population of 11 inbred lines with diverse geographic origins was phenotyped in four consecutive field trials, in a glasshouse, and two independent experiments in the IPK PhenoSphere.
Research sample	Eleven Zea mays inbred lines were chosen to reflect a diverse geographic origin and also a large range of phenotypic variation.
Sampling strategy	The 11 genotypes each grew in two double row plots (three double row plots in 2019) consisting of 18 plants each in the field environments 2016, 2017, and 2018. In both PhenoSphere experiments there were five double row plots per genotype. The double row plots were always grown in a complete randomized plot design. From the inner six individuals of each double row plot, five representative individuals were selected and phenotyped to avoid border effects. In the glasshouse, plants were grown in pots in ten rows, each containing a replicate of the 11 genotypes in randomized order and all plants were phenotyped. The sample size in the field 2016, 2017, and 2018 was 10 replicates per genotype, 15 replicates in 2019, 25 replicates in the PhenoSphere cultivations, and 10 replicates in the glasshouse. The sample sizes were chosen to cultivate maize plants in a field-like density and to maximize the utilization of the available space in each environment.
Data collection	The phenotypic data was recorded by Marc Heuermann, Dominic Knoch, Gunda Wehrstedt, Iris Fischer, Marion Michaelis, Alexandra Rech, Andrea Apelt, Sibille Bettermann, Monika Gottowik, and Beatrice Knüpfer. The data was recorded by measuring plant height with a ruler and noting plant height, vegetative stage, growth stage and time point of tasseling by pen and paper. Grain yield was sampled as the whole cob and weights and grain number were determined in the lab using a lab balance.
Timing and spatial scale	The phenotypic data on the field 2016, 2017, and 2018 was collected twice a week over the whole growing season from April to September in the respective years. In the field 2019 data was collected only once a week from April to September due to limited availability of human resources. In the PhenoSphere and glasshouse data was again collected twice a week. The 'PhenoSphere avg' and glasshouse experiment were conducted in parallel from November 2018 to May 2019. The 'PhenoSphere 2016 sim' experiment was performed from November 2019 to May 2020. Data acquisition was stopped individually per genotype, when all leaves fully matured. The spatial area per field was 351 m^2 with protective border planting, 116 m^2 in the PhenoSphere compartment 1, and 28 m^2 in the glasshouse.
Data exclusions	No data were excluded
Reproducibility	The study tests the capability of the IPK PhenoSphere to simulate field conditions. 11 maize genotypes were tested in 7 environments and for each genotype x environment combination between 10 and 25 individuals were measured.
Randomization	Genotypes were grown in randomized block designs.

Blinding	The measured traits plant height, vegetative stage and growth stage did not require blinding as they are well defined and not subjective.

Did the study involve field work? ☒ Yes ☐ No

Field work, collection and transport

Field conditions	Plants were planted manually and environmental conditions were recorded at a local weather station.
Location	Field sites of the IPK are located directly on the campus. Latitude: 51.826985 | Longitude: 11.272046 Altitude: 120 meters
Access & import/export	The field sites are fenced in and access is restricted to IPK users.
Disturbance	No wildlife was disturbed.

Reporting for specific materials, systems and methods

We require information from authors about some types of materials, experimental systems and methods used in many studies. Here, indicate whether each material, system or method listed is relevant to your study. If you are not sure if a list item applies to your research, read the appropriate section before selecting a response.

Materials & experimental systems

n/a	Involved in the study	
☒	☐	Antibodies
☒	☐	Eukaryotic cell lines
☒	☐	Palaeontology and archaeology
☒	☐	Animals and other organisms
☒	☐	Clinical data
☒	☐	Dual use research of concern
☐	☒	Plants

Methods

n/a	Involved in the study	
☒	☐	ChIP-seq
☒	☐	Flow cytometry
☒	☐	MRI-based neuroimaging

Plants

Seed stocks	Five Zea mays inbred lines from the yellow dent, stiff stalk, and non-stiff stalk breeding pools (B73, N22, P148, PHT77, and S052) and six accessions (amplified after single seed descent, SSD, passage) from the Genebank of the IPK Gatersleben (ZEA 132, ZEA 324, ZEA 332, ZEA 3660, ZEA 399, and ZEA 851)
Novel plant genotypes	Not Applicable
Authentication	Not Applicable
